# Spatial Transcriptomics Unveils the Blueprint of Mammalian Lung Development

**DOI:** 10.1093/gpbjnl/qzaf053

**Published:** 2025-06-10

**Authors:** Mingyue Chen (陈明月), Junjie Lv (吕俊杰), Qiao Zhang (张乔), Qian Gong (龚倩), Ting Zhao (赵婷), Zhenping Chen (陈振平), Haishen Xu (徐海深), Nan Zhou (周南), Shan Jiang (姜姗), Jian Du (都建), Xuepeng Chen (陈雪鹏), Yuwen Ke (柯玉文)

**Affiliations:** State Key Laboratory of Animal Biotech Breeding, College of Biological Science, China Agricultural University, Beijing 100193, China; Guangzhou National Laboratory, Guangzhou 510005, China; State Key Laboratory of Animal Biotech Breeding, College of Biological Science, China Agricultural University, Beijing 100193, China; Guangzhou National Laboratory, Guangzhou 510005, China; Guangzhou National Laboratory, Guangzhou 510005, China; Guangzhou National Laboratory, Guangzhou 510005, China; State Key Laboratory of Animal Biotech Breeding, College of Biological Science, China Agricultural University, Beijing 100193, China; Guangzhou National Laboratory, Guangzhou 510005, China; State Key Laboratory of Animal Biotech Breeding, College of Biological Science, China Agricultural University, Beijing 100193, China; Department of Biochemistry and Molecular Biology, Research Center for Infectious Diseases, School of Basic Medical Sciences, Anhui Medical University, Hefei 230032, China; Key Laboratory of Epigenetic Regulation and Intervention, Institute of Biophysics, Chinese Academy of Sciences, Beijing 100101, China; National Laboratory of Biomacromolecules, Institute of Biophysics, Chinese Academy of Sciences, Beijing 100101, China; Department of Biochemistry and Molecular Biology, Research Center for Infectious Diseases, School of Basic Medical Sciences, Anhui Medical University, Hefei 230032, China; Guangzhou National Laboratory, Guangzhou 510005, China; GMU-GIBH Joint School of Life Sciences, The Guangdong–Hong Kong–Macau Joint Laboratory for Cell Fate Regulation and Diseases, Guangzhou National Laboratory, Guangzhou Medical University, Guangzhou 510005, China; The Fifth Affiliated Hospital of Guangzhou Medical University, Guangzhou Medical University, Guangzhou 510700, China; State Key Laboratory of Animal Biotech Breeding, College of Biological Science, China Agricultural University, Beijing 100193, China; Frontiers Science Center for Molecular Design Breeding (MOE), China Agricultural University, Beijing 100193, China

**Keywords:** Spatial multi-omics, Lung development, Regulation of transcription factor, Cell–cell communication, Alveologenesis

## Abstract

Mammalian lung development is a complex, highly orchestrated process involving the precise coordination of diverse cell types. Despite significant advances, the spatial gene expression patterns and regulatory mechanisms within the developmental niches of different lung structures remain incompletely understood. In this study, we present a comprehensive spatial transcriptomic atlas of mouse lung development, spanning from the early pseudoglandular to the alveolar stage. We further uncovered transcription factor (TF) regulatory landscapes by integrating the spatial epigenome, including novel TF-enhancer-driven regulons (eRegulons) critical for epithelial progenitors during early lung development. Our analysis also identified hundreds of spatiotemporally dynamic cell–cell communications, such as *Bmp8b*-mediated ligand–receptor signaling enriched in airway branching. Notably, we delineated the distinct developmental trajectories of alveolar AT1 and AT2 cells and revealed that collagen pathways facilitated their spatial convergence, forming primary alveoli during the canalicular–saccular transition. Together, this spatial transcriptomic atlas provides a foundational resource for understanding the complex cellular and molecular orchestration underlying mammalian lung development.

## Graphical abstract

**Figure:**
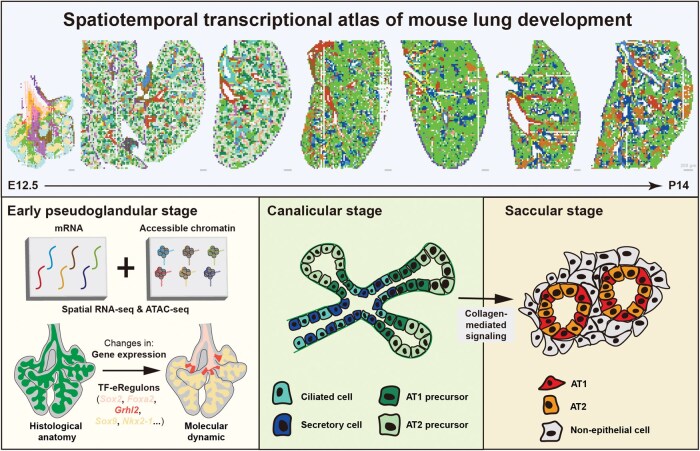

## Introduction

The lung is the primary organ responsible for gas exchange and tissue homeostasis in mammals [1]. Abnormal gene expression during lung development can lead to various respiratory diseases, including congenital pulmonary hypoplasia, pediatric asthma, and adult-acquired lung diseases [2–5]. Mammalian lung development involves a series of morphogenetic processes, such as airway branching, epithelial–mesenchymal interactions, vascularization, interstitial thinning, and alveolar formation [5–7].

Similar to that in humans, mouse lung development consists of five major stages based on morphological criteria: embryonic, pseudoglandular, canalicular, saccular, and alveolar [6]. During the embryonic stage, the two lung buds separate from the foregut, forming the primary lung airways. The airways then elongate and undergo branching morphogenesis during the pseudoglandular stage. During the canalicular and saccular stages, the most distal airways are formed, providing the foundational structure for future gas exchange. Notably, the saccular stage marks the end of branching morphogenesis and is characterized by ongoing alveolar epithelial differentiation, which is crucial for healthy pulmonary function after birth. Finally, alveolarization involves thinning of the alveolar septa and maturation of the microvascular network, culminating in the establishment of mature gas exchange.

Recent single-cell transcriptomic studies have identified various cell types in the developmental and adult lungs of mice and humans [8–14]. However, these studies often lack spatial information about the naïve locations of these cells. Investigating the spatial distribution of gene expression among different lung cell types, including epithelial, endothelial, and mesenchymal cells, is essential for understanding the molecular microenvironments that drive lung development.

Additionally, lung epithelial progenitor cells during the embryonic stage are typically distributed along the proximal–distal axis within the airways [15]. However, the chromatin accessibility landscapes of these critical progenitor cells remain largely unexplored. Furthermore, *in situ* epithelial–mesenchymal interactions within tissues play a vital role in branching morphogenesis and alveolar formation. Yet, the systematic identification of high-fidelity cell–cell communications and their regulatory roles in the formation of complex lung structures has not been fully resolved.

Spatially resolved transcriptomic methods provide both transcriptomic data and positional context for cells within tissues [16, 17]. Leveraging emerging high-resolution spatial transcriptomic technology, deterministic barcoding in tissue for spatial omics sequencing (DBiT-seq), we generated a comprehensive spatial transcriptomic atlas of mouse lung development to explore the dynamic spatial context of various cells and key molecules. We identified representative spatial lung cell clusters and validated the spatial expression distributions of their marker genes. Additionally, we integrated spatial transcriptomic and epigenomic data to reveal the regulatory landscapes of relevant transcription factors (TFs) in lung epithelial progenitor cells. Notably, we also discovered novel spatiotemporal cell–cell communications that elucidate the spatial orchestration of lung development in mice. Overall, this study establishes a valuable spatial omics foundation for enhancing our understanding of mammalian lung development.

## Results

### Spatial transcriptomic atlas of lung development in mice

**Figure 1:**
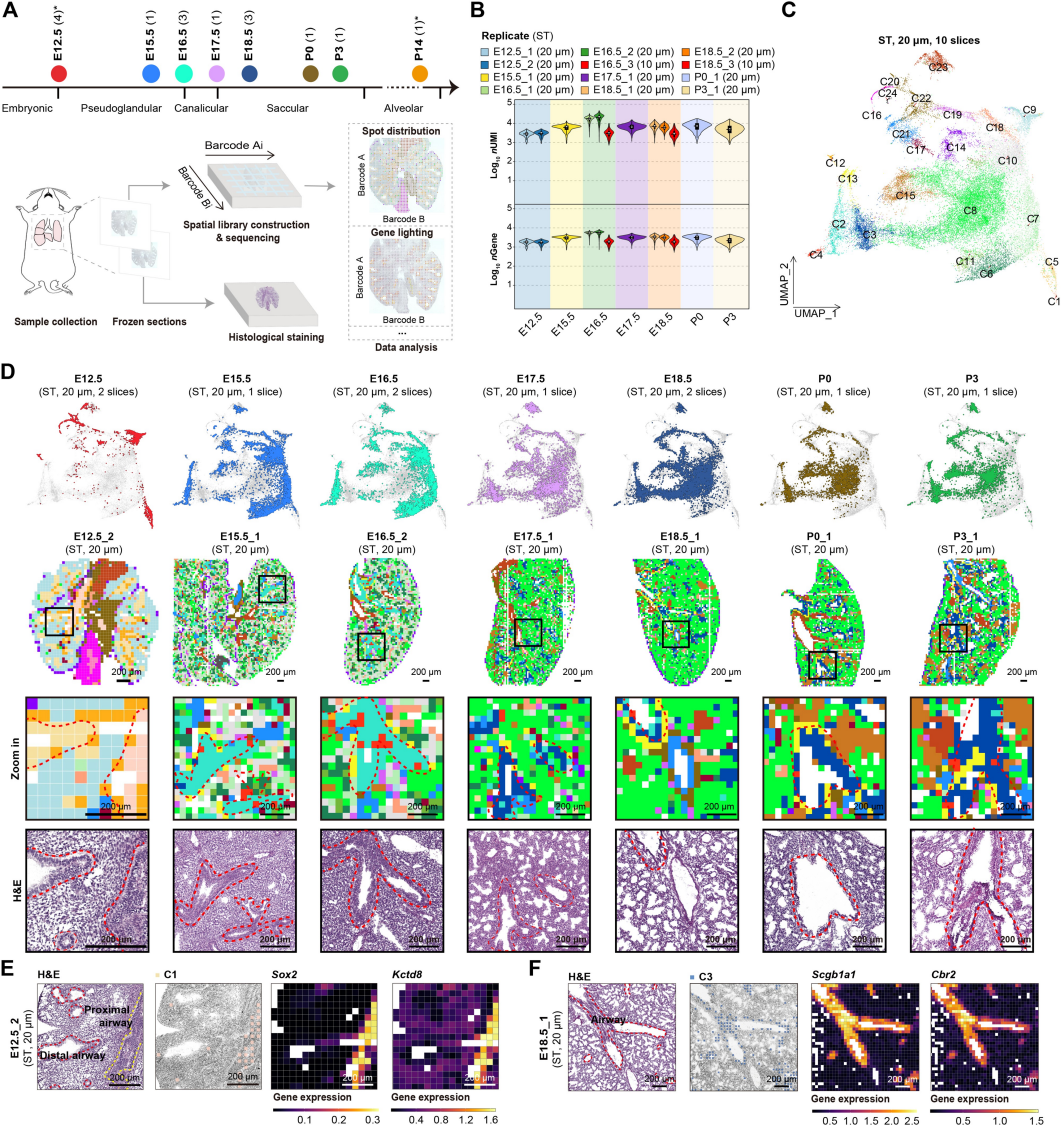
Spatiotemporal transcriptomic atlas of mouse lung development **A**. Schematic workflow for constructing the spatial transcriptomic and epigenomic atlas during mouse lung development. Samples span five morphologically distinct developmental stages. The E12.5 data, marked with an asterisk (*), comprise two ST libraries and two spatial ATAC-seq and RNA-seq libraries. The P14 ST data, also marked with an asterisk (*), were generated using Stereo-seq in this study. The number of biological replicates for each developmental time point is indicated in parentheses. **B**. Violin plots displaying the distribution of log_10 _*n*UMI and log_10 _*n*Gene across samples. The resolution of the ST data is indicated as 20 μm or 10 μm. **C**. UMAP embedding of all modified DBiT-seq profiled spots (20 μm resolution) across E12.5 to P3 stages. **D**. UMAP plots highlighting spots from different developmental time points (row 1). Spatial visualization of one representative section for each time point (row 2). Zoomed-in regions showing specific clusters (row 3). Corresponding H&E staining of the adjacent slices for each section. Airway structures are delineated by red dashed lines (row 4). Scale bars: 200 μm. **E**. Normalized spatial expression of C1 (Sox2^+^ epithelial progenitor cell-enriched) markers: *Sox2* (canonical) and *Kctd8* (newly identified) at E12.5. Red dotted lines indicate distal airways, and yellow dotted lines mark proximal airways. Scale bars: 200 μm. **F**. Normalized spatial expression of C3 (secretory cell-enriched) markers: *Scgb1a1* (canonical) and *Cbr2* (newly identified) at E18.5. Red dotted lines on H&E staining highlight airway structures. Scale bars: 200 μm. ST, spatial transcriptomic; UMAP, uniform manifold approximation and projection; ATAC-seq, assay for transposase-accessible chromatin with high throughput sequencing; RNA-seq, RNA sequencing; Stereo-seq, spatial enhanced resolution omics-sequencing; DBiT-seq, deterministic barcoding in tissue for spatial omics sequencing; *n*UMI, number of unique molecular identifiers; *n*Gene, number of detected genes; E12.5, embryonic day 12.5; P14, postnatal day 14; H&E, hematoxylin and eosin stain.

To systematically characterize spatial gene expression dynamics and cellular interactions during mammalian lung morphogenesis, we established a spatial transcriptomic atlas encompassing embryonic day 12.5 (E12.5) through postnatal day 14 (P14), capturing critical stages of lung development (Figure 1A, Figure S1A) [2]. Spatial transcriptomic (ST) profiling was performed using modified DBiT-seq [18] at 20 μm/10 μm resolution with biological replicates (Figure 1A, Figure S1A; Table S1). We used immunofluorescence (IF) staining to confirm progression of mouse lung development, evidenced by NKX2.1^+^ epithelial localization and ACTA2^+^ smooth muscle differentiation patterns (Figure S1B). Following data preprocessing and quality control, our mouse lung ST atlas encompassed 27,213 genes (Figure 1B; Table S2). The median number of unique molecular identifiers (UMIs) per ST spot was 5365, with a range from 2824 to 21,409 (Figure 1B). The median number of genes detected per spot reached 2758, with a range from 1805 to 5813 (Figure 1B).

Then, dimensionality reduction and unsupervised clustering of all 20 μm resolution ST data (E12.5–P3) via uniform manifold approximation and projection (UMAP) revealed 24 transcriptionally distinct lung spatial clusters, each exhibiting unique molecular signatures and histotopographical distributions (Figure 1C and D, Figure S1C–E; Table S3) (Materials and methods). Cluster-specific marker genes closely matched the spatial localization of each cluster. For example, the canonical epithelial progenitor marker *Sox2* and the novel candidate marker *Kctd8* showed co-localization in the early proximal airway (C1), while *Scgb1a1* (secretory cell marker) and *Cbr2* were specific to bronchial cluster C3 (Figure 1E and F). Additionally, *Wt1* expression characterized mesothelial cluster C14, and *Vegfc* marked vascular endothelial cluster C18 (Figure S1F and G).

Next, computational integration with existing single-cell RNA sequencing (scRNA-seq) references [10,13] via cell2location [19] established high-confidence spatial annotations (Figure S2A–E). Spatial clusters showed predominant enrichment for important lung cell types, including Sox2^+^ epithelial progenitors (C1) and pulmonary neuroendocrine cells (PNECs; C4) (Figure S2C–E). Functional enrichment analysis of 21 out of 24 clusters (7 epithelial, 7 mesenchyme-derived, and 7 vasculature-associated) uncovered cluster-specific biological programs, including ciliated cell specialization through cilium assembly machinery in C2 (Figure S2F).

For the subcellular resolution P14 spatial enhanced resolution omics-sequencing (Stereo-seq) data in this study, we systematically evaluated spatial binning resolutions (10/20/25/50/100 μm) and identified bin100 (50 μm) as the optimal binning strategy, balancing gene detection sensitivity (median 1984 genes/bin100) with spatial coherence (Figure S3A). Unsupervised clustering resolved eight spatial domains, and deconvolution confirmed lung cell-type associations (Figure S3B and C). The P14-C1 domain reflected lung bronchiolar architecture through spatial enrichment of club and ciliated cells (Figure S3C–E). In addition, limited immune cells have been reported in reference atlases [13]. Our deconvolution analysis revealed associations between immune cells and spatial clusters in both DBiT-seq and Stereo-seq datasets (Figures S2D and S3C). However, the widespread distributions of immune cells made it difficult to achieve clear spatial clustering (Figure S3E).

Overall, we generated a comprehensive ST atlas of mouse lung development. Our findings demonstrate that this developing mouse lung ST atlas accurately reveals lung cell diversity and spatial gene expression patterns.

### Modified microfluidic indexing-based spatial assay reveals TF networks in early developing mouse lungs

ST profiling at 20 μm resolution (E12.5_1 and E12.5_2) delineated the proximal–distal segregation of epithelial progenitors (ST-C1: proximal-enriched; ST-C2: distal-enriched), which was also supported by single-cell transcriptomic deconvolution (Figure S4A–E). To elucidate the epigenetic regulatory landscape governing this spatial diversification of epithelial progenitors, we implemented a modified microfluidic indexing-based spatial assay for transposase-accessible chromatin and RNA-sequencing (MISAR-seq) platform [20] at 10 μm resolution for simultaneously mapping chromatin accessibility and gene expression in E12.5 lungs [spatial multi-omic (SMO) data] (Figures S5A–F and S6A and B).

Chromatin accessibility profiling of E12.5 lungs demonstrated robust technical metrics, with 17,635 median high-quality fragments per spot for E12.5_3 and 19,339.5 fragments for E12.5_4 (Figure S5A and B; Table S2). Besides, we detected about 3000 genes per spatial spot in the E12.5 SMO data (Figure S5A; Table S2). Furthermore, unsupervised clustering of chromatin accessibility [assay for transposase-accessible chromatin with high throughput sequencing (ATAC-seq)-only, 9 clusters], transcriptome [RNA sequencing (RNA-seq)-only, 12 clusters], and integrated modalities (ATAC-seq and RNA-seq, 11 clusters) revealed a strong inter-modality concordance (Figure S5C–F) (Materials and methods).

**Figure 2:**
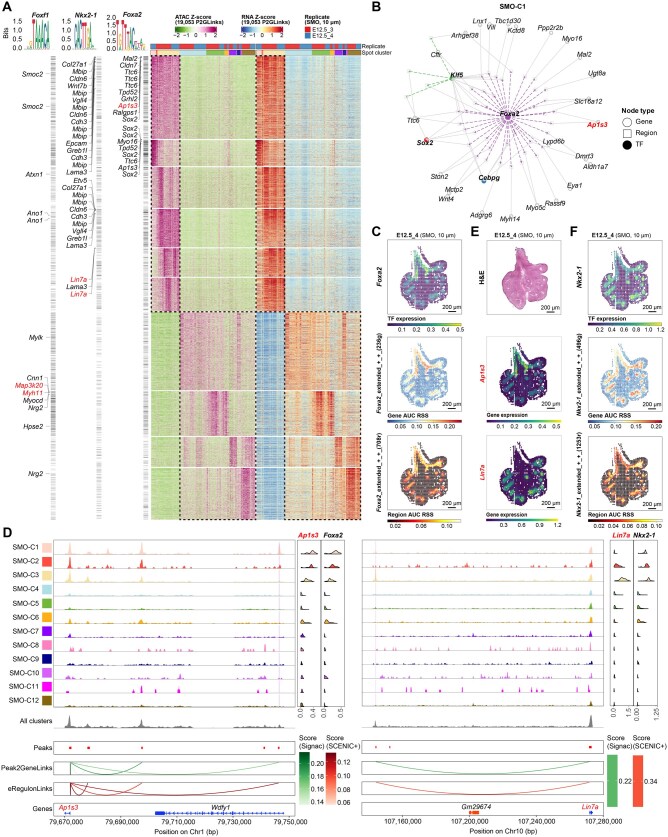
Spatial transcriptome and epigenome of the early developing mouse lung **A**. Heatmap depicting chromatin accessibility and gene expression for 19,053 significant peak–gene linkages with E12.5_3 and E12.5_4 SMO data. Each row represents a peak–gene pair, with individual peaks potentially linked to multiple genes and vice versa. Peaks containing motifs for *Foxa2*, *Nkx2-1*, and *Foxf1* TFs are annotated on the left. **B**. TF-region–gene regulatory network specific to SMO-C1. Dashed lines denote TF-region regulatory relationships, while solid lines represent region–gene regulatory interactions. **C**. Spatial mapping of *Foxa2* TF-eRegulon specific to spatial cluster SMO-C1 (Sox2^+^ epithelial progenitor-enriched), visualized through TF expression, gene AUC RSS, and region AUC RSS. Scale bars: 200 μm. **D**. Genome browser tracks illustrating chromatin accessibility (top), peak loci, Signac-derived Peak2GeneLinks, SCENIC+ predicted eRegulonLinks (middle), gene tracks (bottom), and corresponding gene expression (right panel) for TF-eRegulon–target gene pairs: *Foxa2*–*Ap1s3* (left) and *Nkx2-1*–*Lin7a* (right). **E**. H&E staining of the adjacent slice of E12.5_4 and spatial visualization of target genes *Ap1s3* and *Lin7a* on E12.5_4, demonstrating their preferential localization along proximal and distal airway structures, respectively. Scale bars: 200 μm. **F**. Spatial mapping of *Nkx2-1* TF-eRegulon specific to SMO-C3 (Sox9^+^ epithelial progenitor-enriched), displayed via TF expression, gene AUC RSS, and region AUC RSS. Scale bars: 200 μm. SMO, spatial multi-omic; TFs, transcription factors; AUC, area under the recovery curve; RSS, cell-type specificity; SCENIC+, single-cell regulatory network inference and clustering plus.

Further integrative analysis identified 19,053 association links between chromatin accessibility peaks and target genes (P2G links), with associated heatmaps confirming the concordance for P2G links between chromatin accessibility and gene expression (Figure 2A). We identified *Foxa2*, *Nkx2-1*, and *Foxf1* as principal transcriptional regulators through TF motif enrichment analysis, and subsequently mapped their binding sites across all P2G links (Figure 2A). While confirming the known regulatory interaction between *Etv5* and *Nkx2-1* in epithelial populations [21], our analysis further identified novel associations involving *Ap1s3* with *Foxa2* motifs and *Lin7a* with *Nkx2-1* motifs (Figure 2A). In parallel, both *Myh11* and *Map3k20* showed enrichment in non-epithelial compartments for the *Foxf1* TF motif (Figure 2A).

Furthermore, single-cell regulatory network inference and clustering plus (SCENIC+) TF regulatory module analysis, which can integrate TFs, enhancers, and target genes [22], recapitulated known morphogenic circuits [*Foxa2-*, *Gli1-*, and *Gata6-*enhancer-driven regulons (eRegulons)] [23–25] while uncovering 28 epithelial progenitor-specific TF-eRegulons (Figure 2B, Figure S6C–E). Spatial activation patterns mirrored important developmental knowledge: *Sox2-e*Regulon dominated proximal epithelial progenitors (SMO-C1), whereas *Etv5*/*Sox9*/*Nkx2-1* eRegulons characterized distal epithelial progenitors (SMO-C3) (Figure 2B, Figure S6B and C) [2]. Notably, we discovered many intriguing TF-eRegulons, such as the *Hnf1b, Grhl2*, and *Foxa2*, which were enriched in epithelial progenitors (Figure 2B, Figure S6B–E). *Hnf1b*, previously identified in human late tip epithelial cells [26], exhibited high gene expression in mouse SMO-C2 (stalk epithelial progenitors-enriched) (Figure S6B–D). We also identified novel TF-eRegulons, such as *Rarb* in SMO-C1 and *Rbpjl* in SMO-C3 (Figure S6C and E). *Rarb*, a retinoic acid (RA) nuclear receptor, has been implicated in regulating pulmonary function [27], while *Rbpjl* is known to repress *Notch* target gene expression [28].

Extensive research has established the roles of TFs in cell differentiation, niche formation, and regeneration during mouse lung development [2,29]. Thus, we established a TF-enhancer–target gene regulatory paradigm to understand the regulatory roles of TFs in lung development. For example, *Foxa2*-eRegulon coordinated proximal/stalk progenitor maintenance (SMO-C1/C2), directly occupying *Ap1s3* enhancers to drive airway proximal–distal expression gradients (Figure 2C–E, Figure S7A and B). Similarly, we demonstrated the region-specific expression of *Nkx2-1* TF-eRegulon in distal progenitors (SMO-C3) (Figure 2D and F, Figure S7C). *Nkx2-1* is critical for normal morphogenesis and activates genes specific to pulmonary differentiation, including *Sftpa*, *Sftpb*, and *Sftpc* [30]. We also identified *Lin7a*, a target gene of *Nkx2-1*, which was concentrated in SMO-C3, filling the distal airway (Figure 2D and E, Figure S7B). *Lin7a* is recognized as a critical gene in the onset and progression of various diseases [31–34], suggesting a new role for *Lin7a* in early mouse lung development, potentially regulated by *Nkx2-1* binding to its enhancer (Figure 2D).

*Grhl2* is known to play a substantial role in lung branching morphogenesis, with alterations in *Grhl2* linked to bronchopulmonary dysplasia (BPD), a severe complication in premature infants [35,36]. We observed markedly enhanced expression of *Grhl2* TF-eRegulon in proximal progenitors and stalk epithelial cells (SMO-C1/C2) (Figures S6C and S7C). The expression levels of *Sox2*, *Foxa1*, and *Grhl2* were significantly enriched in the two clusters, consistent with previous findings (Figure S7E) [37,38]. Furthermore, we identified *Sox2*, a well-known marker of lung proximal progenitor cells, as a target gene of *Grhl2* TF, highlighting the cooperative function of cell-specific TF-eRegulons in cell fate determination and maintenance (Figure S7B and E).

In addition to epithelial cells, we also explored TF regulons in mesenchymal and endothelial clusters (Figure S8A). As for non-epithelial cells, our analyses revealed canonical *Wt1* enriched in mesothelial SMO-C7 (Figure S8A). Additionally, we also identified novel regulators, like *Etv1* in proliferating matrix fibroblasts (SMO-C4) and *Zbtb20* in airway smooth muscle cells (ASMCs; SMO-C6) (Figure S8A and B). The TF-eRugulon and peak patterns of *Foxf1* were significantly enriched around the proximal airway (Figure S8C). We further identified the *Foxf1*–*Map3k20* regulatory axis, with the *Foxf1* motif located in the enhancer region of *Map3k20* (Figure S8D). Notably, the spatial visualization of *Map3k20* mirrored the pattern of *Foxf1* TF (Figure S8C and E). Finally, we demonstrated the specific expression of *Map3k20* and *Foxf1* in mesenchymal SMO-C6 (Figure S8D), while the MAPK pathway in the lung mesenchymal layer has been shown to coordinate effective epithelial–mesenchymal interactions during lung development [39].

Our integrated SMO data have systematically reconstructed the regulatory blueprint of pulmonary morphogenesis, revealing cell type-specific TF-eRegulon networks comprising master TF regulators, lineage-defining *cis*-regulatory elements, and functionally validated target genes in early mouse lungs.

### Spatial cell–cell communications in developing mouse lungs

During lung development, cellular organization establishes distinct anatomical compartments, including airway, vascular, and mesenchymal niches. These spatially defined microenvironments and their associated signaling cascades critically regulate cellular fate determination and differentiation trajectories [40–42]. To systematically map intercellular communication networks, we employed SpatialDM, a computational framework specifically designed for ST analysis that incorporates spatial autocorrelation metrics [43], and identified hundreds of significant ligand–receptor (LR) interactions within developing lung niches (Figure S9A; Table S4). The calculated Moran’s I values for these LR pairs demonstrated a strong positive correlation between biological replicates with Pearson’s *r* > 0.88 (Figure S9B). Introducing non-lung ST data from melanoma and intestine further revealed that LR pairs identified in the mouse lung displayed a clustered pattern distinct from that of melanoma and intestine ST data (Figure S9C).

**Figure 3:**
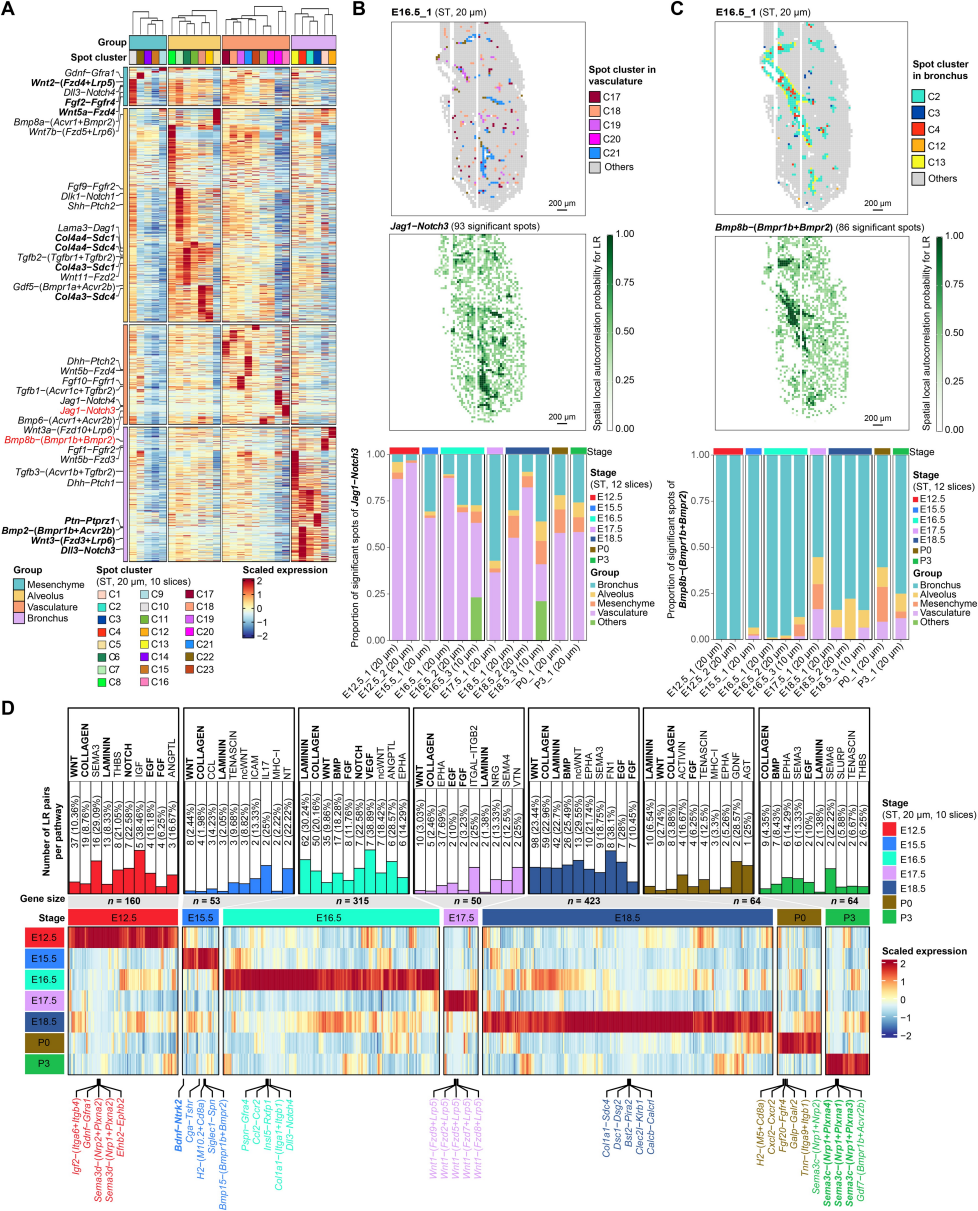
Spatial cell–cell communications in the developing mouse lung **A**. Heatmap depicting lung structure-specific cell–cell communication dynamics during mouse lung development. All 20 μm resolution ST spots were stratified into four anatomical groups: mesenchyme, alveolus, vasculature, and bronchus, based on structural localization. **B**. Spatial distribution of spot clusters within the vasculature group (top). Spatial local autocorrelation probability (1–z_pval) map for *Jag1*–*Notch3* interactions (middle). The histogram showing the proportion of *Jag1*–*Notch3* across different lung structure groups (bottom). Scale bars: 200 μm. **C**. Spatial distribution of spot clusters within the bronchi group (top). Spatial local autocorrelation probability (1–z_pval) map for *Bmp8b*–(*Bmpr1b*+*Bmpr2*) interactions (middle). The histogram showing the proportion of *Bmp8b*–(*Bmpr1b*+*Bmpr2*) interactions across lung structural groups (bottom). Scale bars: 200 μm. **D**. Heatmap illustrating temporally dynamic LR during lung development. Overlaid bar plots (top) indicate the pathway-specific distribution of identified LR interactions. LR, ligand–receptor pair; *Jag1*, Jagged 1; *Notch3*, Notch receptor 3; Bmp, bone morphogenetic protein.

Spatiotemporal analysis revealed distinct LR enrichment patterns across developing lung anatomical compartments, including mesenchyme, bronchus, alveolus, and vasculature (Figure 3A). Previously reported LR pairs associated with Wnt, Notch, and FGF signaling pathways [3,44–46] exhibited compartment-specific enrichment. For instance, *Fgf2*–*Fgfr4*, *Wnt2*–(*Fzd4*+*Lrp5*), and *Wnt5a*–*Fzd4* were significantly enriched in the lung mesenchyme, while *Bmp2*–(*Bmpr1b*+*Acvr2b*), *Wnt3*–(*Fzd3*+*Lrp6*), and *Dll3*–*Notch3* were more abundant in the bronchus (Figure 3A). *Ptn*–*Ptprz1* signaling was demonstrated with distinct enrichment in airway structures (Figure 3A), supporting PTN’s role in normal lung airway development [47]. We also observed *Jag1*–*Notch3* enrichment in vascular structures [48] (Figure 3A and B).

Furthermore, our analysis uncovered multiple novel LR interactions during mouse lung development. Bone morphogenetic proteins (BMPs), like BMP2, BMP3, BMP4, BMP5, BMP6, and BMP7, are known to regulate mammalian lung development [49]. Notably, we identified *Bmp8b*-mediated signaling as a novel regulator of airway morphogenesis, expanding its known roles in spermatogenesis and thermogenesis [50–52]. Spatial mapping revealed *Bmp8b*–(*Bmpr1b*+*Bmpr2*) and *Bmp8b*–(*Bmpr1b*+*Acvr2b*) enrichment in the airway, particularly at branching points in the bronchus (Figure 3A and C, Figure S9D). The enrichment of *Bmp8b*–(*Bmpr1b*+*Acvr2b*) in bronchial structures maintained a similar enrichment pattern during lung development, spanning from E12.5 to P14 (Figure S9D).

In addition, our temporal analysis for LR signaling also revealed stage-specific LR pairs. *Bdnf*–*Ntrk2* was specifically enriched in the early stages of lung development, while *Sema3c*–*Nrp/Plxn* LR pairs were enriched postnatally (P3) (Figure 3D). These LR pairs were enriched not only in canonical pathways (Wnt, FGF, BMP, and Notch) but also in collagen and laminin signaling (Figure 3D), implicating extracellular matrix remodeling in developmental regulation. Lung compartment enrichment change was observed for *Col1a2*–(*Itga11*+*Itgb1*), which transitioned from vascular-specific enrichment (E12.5) to dual airway–vascular distribution (E15.5–P3) (Figure S9E), suggesting its dual roles in vascular maturation and airway morphogenesis.

This comprehensive spatial LR analysis reveals hierarchical and dynamic LR signaling orchestrating pulmonary morphogenesis through known pathways and novel intercellular interactions. The spatiotemporal enrichment redistribution of LR pairs demonstrated the plasticity of cell–cell communication during lung development, offering new insights into developmental signaling.

### Temporal development of lung conducting airway and vascular cells

To delineate the spatial organization of pulmonary conducting airways, we performed sub-clustering analysis on 2233 spatially resolved airway spots from developing mouse lungs (Figure S10A and B) (Materials and methods). This analysis identified five distinct transcriptional clusters: AW-C1 (Sox2^+^ early epithelium-enriched), AW-C2/C3 (secretory/ciliated cell-enriched), AW-C4 (neuroendocrine cell-enriched), and AW-C5 (ASMC-enriched) (Figure S10C). Spatial mapping confirmed the compartment-specific expression of canonical markers: *Spag17* (ciliated cells), *Scgb3a2* (secretory cells), *Pcsk1* (neuroendocrine cells), and *Myh11* (ASMCs) within conducting airway structures (Figure S10D). The temporal comparison revealed dynamic shifts in proportions among these epithelial subpopulations throughout pulmonary morphogenesis (Figure S10E). PNECs, a rare yet functionally critical epithelial population, play essential roles in pulmonary physiology through bioactive amines and neuropeptide production, with established links to asthma pathogenesis and small cell lung cancer development [53–55]. Our pseudotime analysis showed that PNECs emerged very early in the conducting airway (Figure S10F and G), which is consistent with previous reports [56,57].

Vascular compartment analysis identified six vasculature-associated subclusters, such as arterial endothelial cells (AECs)-enriched EC-C2 and pericyte-enriched EC-C5 populations (Figure S11A and B). Molecular characterization showed *Trpc6* is a definitive pericyte marker (EC-C5) and *Kit* is a general capillary (gCap) signature (EC-C6) (Figure S11C). Near-cellular resolution spatial mapping (10 μm) revealed scattered expression of *Kit* and *Trpc6* in regions outside large airways and vascular structures at 10 μm resolution E18.5 ST sections (Figure S11D).

In summary, our ST analysis delineates the molecular characteristics of conducting airway and vascular cells in mouse lungs, confirming PNECs as an early-specified epithelial lineage during pulmonary morphogenesis.

### Divergent developmental trajectories of alveolar epithelial lineages

**Figure 4:**
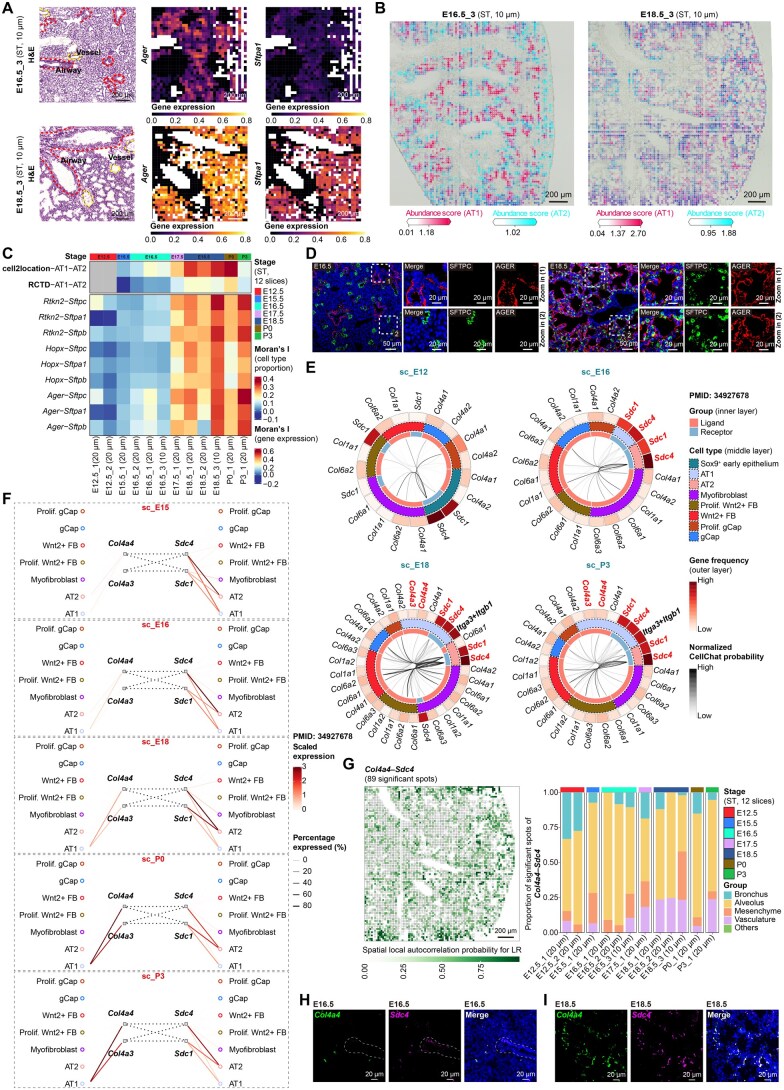
Spatial convergence of AT1 and AT2 cells and alveolar niche formation **A**. High-resolution (10 μm) ST profiles of AT1 marker *Ager* and AT2 marker *Sftpa1* expression at E16.5 and E18.5. Red dashed lines demarcate airway structures, while yellow dashed lines indicate vascular networks. Scale bars: 200 μm. **B**. Spatial distribution patterns of AT1 and AT2 cells visualized through cell2location abundance scores at E16.5 and E18.5. Scale bars: 200 μm. **C**. Heatmap comparing spatial autocorrelation (Moran’s I) between RCTD weights and cell2location scores for AT1 and AT2 cells, alongside established AT1 (*Rtkn2*, *Hopx*, and *Ager*) and AT2 (*Sftpc*, *Sftpa1*, and *Sftpb*) marker gene pairs. **D**. IF staining of AGER (AT1) and SFTPC (AT2) at E16.5 and E18.5 lungs. Scale bars: 50 μm. The zoomed-in regions are shown on the right. Scale bars: 20 μm. **E**. Circle plot illustrating LR pairs involving distal epithelial cell populations, including Sox9^+^ early epithelium, AT1, and AT2 cells, as receptor-expressing populations in collagen signaling pathways, derived from scRNA-seq data (PMID: 34927678). **F**. Expression probability and level for collagen ligands (*Col4a4*/*Col4a3*) and their receptors (*Sdc4*/*Sdc1*) across alveolar niche cell subtypes in the scRNA-seq data (PMID: 34927678). **G**. Spatial probability map (1 − z_pval) of *Col4a4*–*Sdc4* co-localization (left) with corresponding structural distribution analysis across lung compartments (right). **H**. and **I**. RNA fluorescence *in situ* hybridization confirming the spatial expression of *Col4a4* and *Sdc4* at E16.5 (H) and E18.5 (I), with white dashed lines outlining developing airway structures. Scale bars: 20 μm. RCTD, Robust Cell Type Decomposition; scRNA-seq, single-cell RNA sequencing. IF, immunofluorescence; Prolif. gCap, proliferating gCap; gCap, general capillary; Wnt2+ FB, Wnt2+ fibroblast; Prolif. Wnt2+ FB, proliferating Wnt2+ FB.

The alveolar compartment, comprising specialized epithelial cells, fibroblasts, and capillary networks, constitutes the fundamental gas exchange unit in pulmonary architecture [5]. To elucidate alveolar development, we integrated ST datasets of alveolar cells from E16.5 and E18.5 lungs with Sox9^+^ distal progenitors from E12.5 lungs, identifying eight distinct clusters encompassing alveolar epithelial populations and progenitor states (Figure S12A and B). Spatiotemporal analysis revealed progressive maturation signatures, with AT1 marker *Ager* and AT2 marker *Sftpa1* exhibiting significant gene expression increases during the canalicular-to-saccular transition (**Figure 4**A).

Furthermore, Single-cell Trajectories Reconstruction, Exploration And Mapping (STREAM) pseudotime analysis [58] delineated divergent differentiation paths for alveolar epithelial cells. Sox9^+^ progenitors (AL-C1) were segregated into AT1 precursors (AL-C2) and AT2 precursors (AL-C3) through distinct developmental paths (Figure S12C), supporting the paradigm of AT1/AT2 lineage-independent specification rather than a common progenitor model [8,59–61]. RNA velocity analysis using scVelo [62] independently validated these segregated differentiation paths, while maintaining the plasticity of AT2 cells (AL-C5) to transdifferentiate into AT1 lineages (AL-C4) (Figure S12C and D). This is consistent with previous reports [63,64]. AT1 and AT2 lineage-specific gene signatures were also identified: *Sftpb*, *Sftpc*, *Meg3*, *Lamp3*, and *Etv5* for the AT2 lineage, and *Rtkn2*, *Hopx*, *Ndnf*, *Sema3a*, and *Ager* for the AT1 lineage (Figure S12E and F).

Altogether, our ST data support distinct developmental paths for AT1 and AT2 cells during the canalicular-to-saccular transition in mouse lungs.

### The spatial gathering of AT1 and AT2 cells and alveolar niche formation

In addition to their distinct developmental trajectories, AT1 and AT2 cells exhibited dynamic spatial location reorganization during alveolar morphogenesis. According to 10 μm resolution ST data in E16.5 and E18.5 lungs, our results revealed that AT1/AT2 segregated spatial distributions in E16.5 canalicular stage lungs would transition to a co-localized pattern at E18.5 saccular stage lungs (Figure 4B). Spatial autocorrelation analysis using Robust Cell Type Decomposition (RCTD) and cell2location algorithms demonstrated a progressive AT1–AT2 proximity, evolving from dispersed distributions before E16.5 to coordinated localization from E17.5 onwards (Figure 4C). The correlation analysis of cellular abundance scores confirmed this spatial location reorganization, with a negative correlation at E16.5 shifting to a positive correlation at E18.5 (Figure S13A). IF staining and RNA fluorescence *in situ* hybridization (FISH) using AT1 marker *Ager* and AT2 marker *Sftpc* both confirmed the ST observation of AT2 cell dispersion along airway tips at E16.5 and subsequent alveolar co-localization with AT1 cells by E18.5 (Figure 4D, Figure S13B; Table S5).

The spatial convergence of AT1 and AT2 cells signifies alveolar niche establishment, mediated by specialized cell–cell communication networks. Previous studies have implicated collagen/integrin-mediated adhesion and actin cytoskeleton remodeling in alveolar elongation and mesenchymal migration [65–67]. We integrated ST and scRNA-seq data [10] and identified notably dynamic collagen/integrin signaling pathways orchestrating niche formation and cell communication within alveolar compartments (Figure 3A and 4E, Figure S13C–E), such as *Col1a1*/*2*–(*Itga3*+*Itgb1*) LR pairs in myofibroblast–AT1 communications and *Col4a1*/*2*–(*Itga3*+*Itgb1*) LR pairs in capillary–AT1 communications (Figure 4E, Figure S13C). Additionally, we found *Itga2*/*Itgb1*-mediated LR signaling, such as *Col1a1*/*2*–(*Itga2*+*Itgb1*), *Col4a1*/*2*–(*Itga2*+*Itgb1*), and *Col6a1*/*2*–(*Itga2*+*Itgb1*) LR pairs enriched in mesenchyme–capillary communications (Figure S13C and E).

Notably, *Col4a3*/*4*–*Sdc1* and *Col4a3*/*4*–*Sdc4* LR interactions, which are part of the *Sdc* receptor-mediated collagen pathways crucial for cell adhesion [68], exhibited the enriched specificity between alveolar epithelial cells, but were absent in alveolar endothelial and mesenchymal cell communications (Figure 4E, Figure S13C). scRNA-seq data [10] revealed the temporal upregulation of *Col4a3/4* in AT1 cells (E16–E18), and relatively stable *Sdc1/4* expression in AT2 cells (Figure 4F). ST visualization corroborated these findings (Figure 4G, Figure S13F). We then applied RNA FISH and IF staining, and further validated *Col4a4* and *Sdc4* enrichment in the distal airway at E16.5, indeed, and alveolar structures at E18.5 (Figure 4H and I, Figure S13G and H; Table S5). We thus propose that these type IV collagen-mediated signaling, specifically the *Col4a3*/*4*–*Sdc1*/*4* axis from AT1 to AT2 cells, contribute to the spatial gathering of AT1 and AT2 cells.

This integrated analysis reveals that alveolar AT1 and AT2 cells undergo spatial gathering during the canalicular–saccular transition, which is probably mediated by type IV collagen–*Sdc* receptor signaling.

### Dynamic gene expression of alveolar domains during perinatal transition in mouse

The transition from intrauterine hypoxia to postnatal oxygenation represents a critical phase in mammalian lung development, requiring precise molecular reprogramming to establish gas exchange competence [1,69]. To resolve these underlying molecular adaptations, we performed dynamic gene expression analysis for alveolar ST domains during the perinatal transition, including prenatal stage (E18.5), birth (P0), and early postnatal (P3) stages (Figure S14A) (Materials and methods).

Differential expression analysis revealed progressive downregulation of hypoxia-inducible factor *Hif3a*, which was consistent with diminished hypoxic signaling as autonomous pulmonary oxygenation postnatally (Figure S14B). This temporal gene dynamic is particularly important, given the established role of *Hif3a* in alveolarization and distal epithelial differentiation for maintaining normal alveolar–vascular architecture [70,71]. The downregulated genes further suggested their involvement in *Wnt*-mediated cell signaling and cellular hypoxia response during environmental oxygen adaptation (Figure S14C). Additionally, *Tgfbi*, a known regulator of NF-κB-dependent angiogenesis during early alveolarization [72], exhibited stage-specific induction, transitioning from near undetectable levels at E18.5 to markedly upregulated levels by P3 in alveolar domains (Figure S14B). Functional enrichment analysis connected these temporally upregulated genes to critical biological processes, including epithelial–mesenchymal crosstalk and NF-κB transcriptional activation, highlighting their coordinated roles in alveolar maturation and microvascular development (Figure S14C). ST visualization further confirmed that *Hif3a*, *Fgf10*, *Fn1*, and *Rtkn2* were highly enriched in the prenatal hypoxic alveolar zone, while *Tgfbi* and *Bmpr2* dominated in alveolar regions after birth (Figure S14D and E).

Overall, our results above delineate the transcriptomic transition during perinatal adaptation in mouse lungs.

### Spatiotemporal expressions of transposable elements during mouse lung development

Protein-coding regions constitute merely ∼ 2% of the mammalian genome. Millions of transposable elements (TEs) are interspersed throughout the whole genome, but their functional roles remain enigmatic [73–75]. Although TE activity is predominantly silenced in somatic tissues, certain elements are transcribed in specific biological contexts, such as neural development and early embryogenesis. The spatial expression dynamics of TEs during lung development, however, remain unexplored.

**Figure 5:**
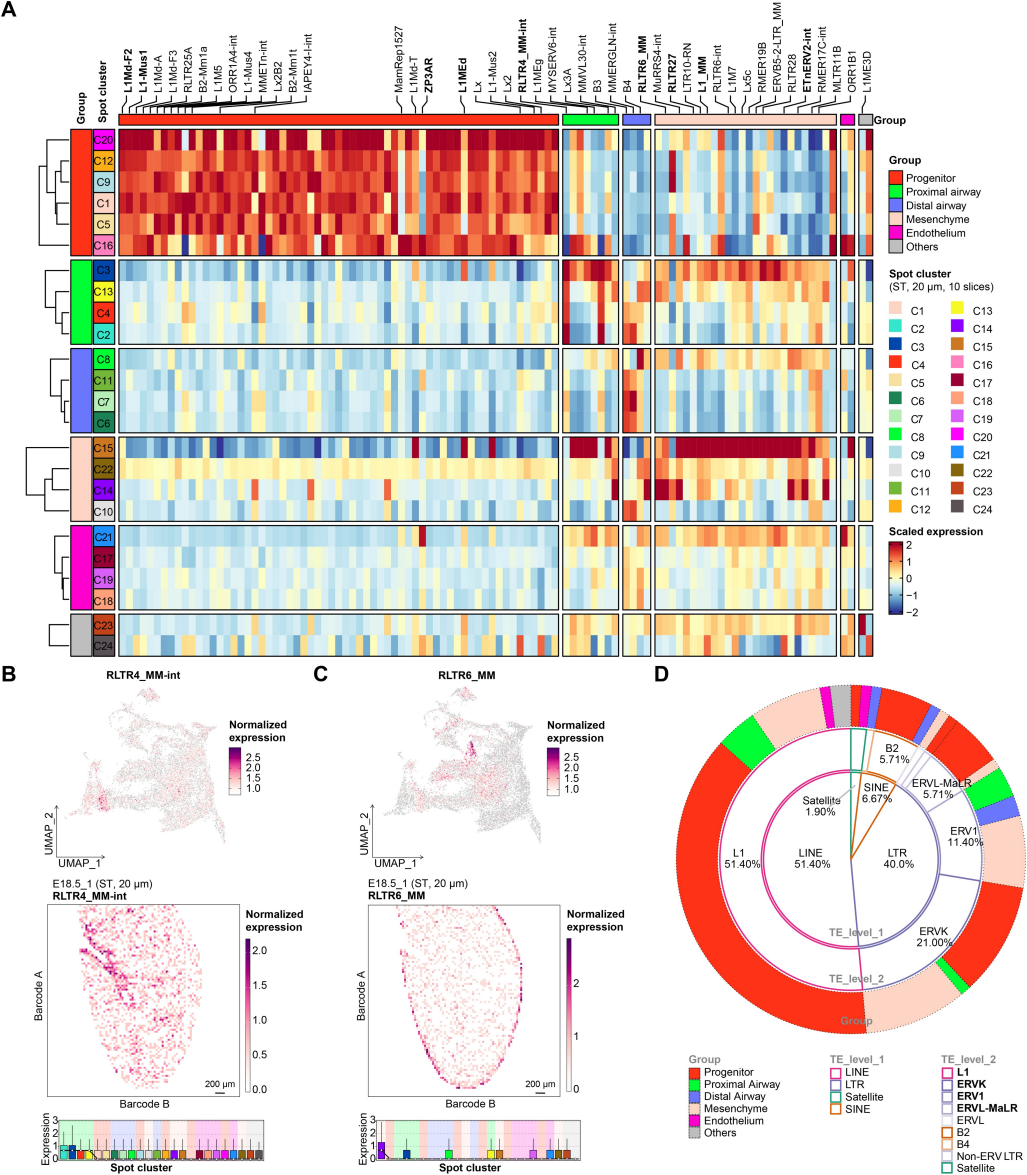
Spatiotemporal expression of TEs during the mouse lung development **A**. Heatmap showing the scaled expression levels of TEs during the mouse lung development in different lung groups. All 20 μm resolution ST spots were divided into six groups based on lung structures and cell states: progenitor, proximal airway, distal airway, mesenchyme, endothelium, and others. **B**. and **C**. UMAP visualization of normalized TE expression levels (top) for RLTR4_MM-int (B) and RLTR6_MM (C). Spatial highlighting of normalized TE expression levels (middle) for RLTR4_MM-int (B) and RLTR6_MM (C). Box plots showing the normalized TE expression levels across different clusters (bottom) for RLTR4_MM-int (B) and RLTR6-MM (C). Scale bars: 200 μm. **D**. Multi-tiered pie chart delineating the compositional distribution of expressed TEs in developing mouse lungs. TE, transposable element; LINE, long interspersed nuclear element; SINE, short interspersed nuclear element; LTR, long terminal repeat. MM, Mus musculu; int, the internal region.

Next, our ST analysis revealed active TE expression across developing lung compartments (**Figure 5**A). Notably, L1 elements like L1Md-F2, L1-Mus1, and L1Med elements exhibited pronounced transcription activity in progenitor populations, including epithelial and endothelial progenitors (Figure 5A). We also observed cluster-specific TE transcription activity patterns. For example, RLTR4_MM-int demonstrated airway epithelial specificity (C2/C3), while RLTR6_MM showed mesothelial enrichment (C14) (Figure 5B and C).

Classification of differentially expressed TEs identified long interspersed nuclear elements (LINEs) and long terminal repeats (LTRs) as the predominant classes (> 90% combined) in the developing mouse lung (Figure 5D). By contrast, short interspersed nuclear elements (SINEs) represented ∼ 7% of active elements (Figure 5D). Specifically, the endothelial clusters exhibited enrichment only for satellite and L1 elements, whereas both proximal and distal epithelium showed co-expression of ERV1 retrotransposons (Figure 5D).

These TE spatial expression analyses indicate that extensive active TE expression exists throughout mouse lung development, with L1 and ERV elements emerging as the predominant classes exhibiting dynamic expressions across different pulmonary structures.

## Discussion

The mammalian lung is a major respiratory organ whose development involves the progressive specialization of diverse cellular lineages through precisely orchestrated morphogenetic processes. This study establishes a comprehensive ST atlas encompassing five critical stages of pulmonary development, providing novel and detailed insights into the spatiotemporal signaling networks governing pulmonary lineage maturation. Building upon recent studies [39,63,76,77], we systematically mapped LR interactions across bronchial, alveolar, vascular, and mesenchymal compartments and delineated cellular responses to developmental signals during lung organogenesis. Beyond canonical Wnt, FGF, and BMP signaling pathways, we identified novel LR interactions, including *Bmp8b*–(*Bmpr1b*+*Bmpr2*) in proximal airways and *Jag1*–*Notch3* in vascular structures, expanding our understanding of pulmonary developmental signaling networks.

Additionally, spatial pseudotime analysis revealed that Sox9^+^ progenitors (AL-C1) diverge into distinct AT1 (AL-C2) and AT2 (AL-C3) precursor populations before maturing into specialized AT1 (AL-C4) and AT2 (AL-C5) lineages. Notably, while AT2 cells initially localized to airway tips at E16.5, they subsequently converged with AT1 cells in alveolar niches by E18.5, a process potentially mediated by *Col4a3*/*4*–*Sdc1* and *Col4a3*/*4*–*Sdc4* interactions, as supported by scRNA-seq, ST data, IF staining, and RNA FISH. Our findings extend previous work demonstrating that AT1 cells are signaling hubs during perinatal maturation [13,67].

While this high-resolution ST and epigenomic atlas provides significant insights, several technical limitations warrant consideration. Current 10 μm resolution spatial-omic platforms cannot achieve real single-cell resolution, resulting in incomplete detection of immune cells and alveolar capillary (aCap) populations that are readily identifiable in parallel single-cell datasets. To address this limitation, we implemented rigorous spatial single-cell integration strategies to improve ST cluster annotations and biological interpretations, complemented by IF and RNA FISH validation. Additionally, the complexity of lung architecture and the limited availability of mesenchyme-specific markers constrain deeper analysis of stromal populations, necessitating higher-resolution spatial technologies and lineage-tracing approaches for comprehensive characterization.

## Conclusion

This study established a comprehensive ST atlas spanning the critical phases of mouse pulmonary development, from late embryonic stages to the alveolar stage. We delineated the TF regulatory networks governing early lung morphogenesis by identifying both novel and known TF-eRegulons. Our analysis identified hundreds of spatiotemporally dynamic cell–cell communications via LR signaling, providing mechanistic insights into lung compartment-specific regulatory programs. Notably, we demonstrated that AT1 and AT2 cells, which originated from separate precursors, underwent spatial convergence through *Col4a3*/*4*–*Sdc1* and *Col4a3*/*4*–*Sdc4* mediated signaling pathways during the canalicular–saccular transition. Additionally, our investigation of TE dynamics revealed the persistent expression of L1, ERVK, and ERVL-MalR elements in progenitor and mesenchymal populations.

Collectively, this study delineates the spatial gene expression landscapes and regulatory networks governing lung compartment-specific development, providing an essential SMO resource for understanding mammalian pulmonary development.

## Materials and methods

### Animals

C57BL/6 background mice were used in this study. Timed-pregnant C57BL/6 females were utilized for all embryonic time points, with pregnancy stages confirmed by crown-rump length measurements. E0.5 was designated as noon on the day a vaginal plug was observed, and P0 was defined as the day of birth. All mice were housed in the Guangzhou National Laboratory animal facility under controlled conditions, including a 12-h light–dark cycle, constant temperature and humidity, and were maintained in a healthy state throughout the study.

### Sample collecting

Given the significant roles of sex and sex hormones in lung development under both physiological and pathophysiological conditions, this study exclusively utilized male mice unless otherwise specified [78–81]. The sex of postnatal mice was determined visually, while polymerase chain reaction (PCR) was employed for sex determination in embryos. PCR was performed using the following primers: Sry-F (5′-ATGAATGCATTTATGGTGTGGTCCCGTGGTGAGAG-3′) and Sry-R (5′-GAGTACAGGTGTGCAGCTCTACTCCAGTCTTGCCT-3′). Fresh frozen lung tissues were collected at E12.5, E15.5, E16.5, E17.5, E18.5, P0, P3, and P14 for ST experiments. E12.5 tissues were used for modified MISAR-seq (10 μm resolution) and DBiT-seq (20 μm resolution), while P14 tissues were exclusively used for 500 nm resolution Stereo-seq library construction. Notably, for P14 samples, blood was removed from the eyes after anesthesia, followed by lung perfusion with 50% optimal cutting temperature compound (OCT) (Catalog No. A4583, SAKURA, Torrance, CA) in phosphate buffer saline (PBS) (v/v) and embedding in OCT. The embedded tissues were immediately stored at −80°C until sectioning for subsequent experiments.

### Microfluidic device acquisition

Fifty 20 μm-wide and ninety-six 10/20 μm-wide polydimethylsiloxane (PDMS) microfluidic chips, along with their matched clamps, custom unembedded slide glass (84.9 × 46.54 × 1 mm), and corresponding storage boxes, were procured from ZhongXinQiHeng (Suzhou CChip Scientific Instrument Co., Ltd., Suzhou, China). The channel interval thickness of the chips was approximately 10 μm for 10 μm-wide chips and 20 μm for 20 μm-wide chips, respectively. Custom poly-L-lysine (Catalog No. P8920-500ML, Sigma-Aldrich, St. Louis, MO) coated glass slides were prepared following the manufacturer’s instructions.

### Tissue cryosection

Prior to sectioning, frozen embedded tissues and slides were equilibrated in the cryostat for at least 30 min to reach the cryostat temperature (∼ −20°C). Tissue sections of 10 μm thickness were prepared for the 20 μm-wide microfluidic channel devices, while 8 μm sections were used for the 10 μm-wide devices. Sections were carefully positioned at the center of pre-marked commercial or custom glass slides, depending on tissue size. During the sectioning process, approximately ten tissue sections were collected into 1.5 ml centrifuge tubes for subsequent RNA quality assessment.

### RNA quality identification

RNA was extracted using the Quick-RNA™ Microprep Kit (Catalog No. R1050, Zymo Research, Irvine, CA) following the manufacturer’s protocol. RNA quality was assessed through agarose gel electrophoresis. Only sections meeting the RNA quality criteria were selected for subsequent spatial transcriptomic experiments.

### Hematoxylin and eosin staining

To assess the histological morphology, adjacent tissue sections from the spatial experiment were subjected to hematoxylin and eosin (H&E) staining using a H&E Staining Kit (Catalog No. C0105S, Beyotime, Shanghai, China). Briefly, air-dried slides were washed with 1× PBS and fixed with 10% neutral-buffered formalin (NBF) for 10 min. After rinsing with distilled water, tissues were stained with hematoxylin solution for 2–4 min and immediately rinsed with distilled water. The sections were then treated with a hydrochloric acid differentiation solution for approximately 10 s. Following another wash, the tissues were counterstained with eosin for about 8 min. Excess eosin was removed using 75% ethanol after a final rinse with distilled water. Slides were air-dried and mounted with coverslips. Stained tissues were imaged using the EVOS™ M7000 Imaging System (Catalog No. AMF7000, Thermo Fisher Scientific, Carlsbad, CA) at 200× magnification.

### Transposome embedding for spatial transcriptome library construction

Unmodified Tn5 transposase was purchased from Novoprotein (Catalog No. M045-01B, Suzhou, China). The transposome complex was assembled according to the manufacturer’s instructions using TN5_Read1_RNA and TN5_ME_RNA adapters (sequences provided in Table S1).

### Spatial transcriptome library construction with modified DBiT-seq

Spatial RNA-seq library construction was performed based on the principle of DBiT-seq [18] with minor modifications.

Briefly, tissue sections were initially cleaned with PBS plus RNase inhibitor (PBS-RI), fixed with 4% formaldehyde (FA), and permeabilized with 0.05% Triton X-100 for 20 min at room temperature. Reverse transcription was then performed by incubating the sections with the reverse transcription (RT) primer at room temperature for 30 min, followed by 42°C for 90 min. Spatial barcoding of messenger RNAs (mRNAs) was achieved using two rounds of PDMS microfluidic chips. Annealed barcode A or B mixtures, blocking A or B mixtures, and 1× NEB buffer 3.1 were sequentially added to the corresponding chip inlets and flowed across the tissue surface through the channels via vacuum. Reverse crosslinking was performed using lysis solution containing proteinase K, and the lysate was collected and stored at −80°C until further processing.

Lysate purification was carried out using the DNA Clean and Concentrator Kit (Catalog No. D4013, Zymo Research) according to the manufacturer’s instructions. Complementary DNA (cDNA) was captured using MyOne™ Streptavidin C1 Beads (Catalog No. 65001, Thermo Fisher Scientific). After washing with B&W buffer (5 mM Tris-HCl pH 8.0, 1 M NaCl, 0.5 mM EDTA) and STE solutions (5 mM Tris-HCl pH 8.0, 25 mM NaCl, 0.5 mM EDTA), the beads were resuspended in template switch mix containing template switch oligo (TSO). Following template switching, the beads were washed with STE and nuclease-free water (NF-H_2_O), resuspended in PCR mix with cDNA_ampli_R and RNA_PCR_primer, and amplified for 10–13 cycles. Amplified products were purified using 0.8× SPRIselect Beads (Catalog No. B23318, Beckman, Brea, CA). cDNA quantity was quantified using a Qubit 4.0 Fluorometer (Thermo Fisher Scientific). For each sample, 50 ng of cDNA was fragmented in a 20 μl tagmentation reaction with custom Tn5 transposase, and the tagmented products were purified using 2× SPRIselect Beads. The 3’ end products were amplified with Illumina Nextera N5 and N7 Adapters and purified with 0.7× SPRIselect Beads. After quality assessment, libraries were sequenced on the NovaSeq 6000 platform (Illumina, San Diego, CA) with 150-bp paired-end reads. Oligonucleotide sequences used in this protocol are listed in Table S1.

### Stereo-seq

P14 lung tissue blocks were prepared and sectioned as described above. Stereo-seq libraries were constructed following previously established protocols [82]. Briefly, P14 lung samples were cryosectioned at a thickness of 10 μM after equilibration in the cryostat for 30 min. The first section was mounted onto the Stereo-seq chip surface, and the subsequent four sections were used to determine the optimal permeabilization time, which was determined to be 6 min. Library construction was performed according to standard procedures, and paired-end 100 bp (PE100) sequencing was conducted.

### Spatial assay for ATAC and RNA-seq with modified MISAR-seq

Spatial ATAC-seq library construction was performed based on the principle of MISAR-seq [20] with minor modifications. Briefly, fresh E12.5 mouse lung sections were subjected to fixation, permeabilization, and transposition. Custom Tn5 transposase, provided by Novoprotein, was used for transposition, along with adapters (ATAC_Read2, ATAC_Read1, and ATAC_ME), as listed in Table S1. Reverse transcription, ligation, and lysis reactions were carried out following the same procedures as described for spatial transcriptome library construction. MyOne™ Streptavidin C1 beads were used to separate cDNA and genomic DNA, and cDNA library construction was performed as outlined above.

For ATAC library preparation, genomic DNA retained in the supernatant after C1 bead separation was purified using the Zymo DNA Clean & Concentrator-5 Kit and eluted in 21 μl of NF-H_2_O. The eluted product was mixed with PCR solution (2.5 μl of 10 μM Illumina Nextera N5 adapters, 2.5 μl of 10 μM Illumina Nextera N7 adapters, and 25 μl of 2× NEBNext Master Mix). PCR amplification was performed using the following program: 72°C for 5 min, 98°C for 30 s, followed by 9 cycles of 98°C for 10 s, 65°C for 30 s, and 72°C for 1 min, with a final extension at 72°C for 5 min. PCR products were purified using 1.2× SPRIselect Beads and resuspended in 20 μl of RNase-free water for sequencing.

### IF staining

P14 and adult male mice were anesthetized with isoflurane and perfused with PBS through the right ventricle. Lungs were inflated with 10% NBF and fixed in 10% NBF at 4°C overnight. Fixed lungs were cryoprotected in PBS containing 20% sucrose and 33% OCT at 4°C overnight, followed by embedding in OCT. For embryonic time points, lungs were dissected from timed-pregnant mice and fixed in 10% NBF at 4°C overnight. Tissues were then gradiently dehydrated in 10%, 20%, and 30% sucrose solutions in PBS, embedded in OCT, and stored at −80°C until further processing.

IF staining was performed using the following primary antibodies: NKX2.1 (Catalog No. WRAB-1231, Seven Hills Bioreagents, Cincinnati, OH; 1:200), ACTA2 (Catalog No. MA1-06110, Thermo Fisher Scientific; 1:250), AGER (Catalog No. MAB1179-SP, R&D Systems, Minneapolis, MN; 1:200), SFTPC (Catalog No. ABC99, Sigma-Aldrich; 1:200), SDC4 (Catalog No. 11820-1-AP, Proteintech, Wuhan, China; 1:100), and COL4A4 (Catalog No. 19674-1-AP, Proteintech; 1:100). Alexa Fluor secondary antibodies included anti-mouse 488 (Catalog No. 4408S, Cell Signaling Technology, Beverly, MA), anti-rat 555 (Catalog No. 4417S, Cell Signaling Technology), anti-rabbit 546 (Catalog No. A-11010, Thermo Fisher Scientific), and anti-rabbit 647 (Catalog No. ab150075, abcam, Cambridge, UK). Images were acquired using a laser confocal microscope (LSM 980, Zeiss, Oberkochen, Germany) with 10×, 20×, or 40× water-immersion objectives at random fields.

### RNA FISH

Target-specific oligonucleotide probes (20–50 nt) conjugated with fluorophores (*e.g.*, Cy3, Alexa 488) were designed and synthesized by Zoonbio Biotechnology Co., Ltd, and frozen tissue sections (10 μm thickness) from E16.5 and E18.5 lungs were prepared for RNA FISH experiments. Briefly, after washing with PBS, tissue sections were treated with proteinase K working solution at 37°C for 20 min. Sections were then incubated with prehybridization solution at 37°C for 1 h to block nonspecific binding. Subsequently, diluted probes were applied to the sections and hybridized overnight at 42°C in a humidified chamber. Following hybridization, sections were washed with gradient saline sodium citrate (SSC) buffers to remove unbound probes. Nuclei were counterstained with 4′,6-diamidino-2-phenylindole (DAPI), and slides were mounted using an anti-fade mounting medium to preserve fluorescence. Imaging was performed using a Nikon inverted fluorescence microscope, and images were acquired for downstream analysis.

### Spatial RNA data pre-processing and quality control

To obtain get gene–spot count matrices, we first trimmed adapters of sequence reads using Cutadapt (v4.2) [83], and then used UMI-tools (v1.1.2) [84] to debarcode. For the final counting step, we utilized zUMIs (v2.9.7d) [85] to obtain UMIs for each spot based on the reference genome (GRCm38) and the corresponding gene transfer format (GTF) file (Ensemble release 102).

To ensure the validity of spots captured by sequencing, we discarded any spot that was not covered on the tissue when mapping to the FIX image (imaging fixed tissue sections during ST library construction). To filter low-quality spots, we excluded spots with fewer than 500 genes detected or 1000 UMIs before later analyses. Additionally, spots with a high level of blood-associated genes were excluded, and this cutoff varied within specific slices. All statistical analyses in violin plots and box plots were produced using wilcox.test(alternative = “two.sided”) of R package stats.

### Stereo-seq data pre-processing and quality control

The raw FASTA + Quality (FASTQ) data for Stereo-seq analysis were processed using the publicly available Stereo-seq Analysis Workflow (SAW) pipeline (v5.4.0) (https://github.com/STOmics/SAW), mapping to the reference genome (GRCm38) and the corresponding GTF (Ensemble release 102). After that, we proceeded with the gene expression profile matrices within non-overlapping bins of 20 × 20, 40 × 40, 50 × 50, 100 × 100, and 200 × 200 DNA nanoballs (DNBs), respectively. We selected bin100 (50 µm) resolution for further analysis and filtered low-quality spots, following the protocol previously described.

### Spatial RNA data normalizing, integrating, clustering, and annotation

We normalized each raw gene–spot matrix individually by SCTransform [86] with the parameters (vst.flavor = “v2”, method = “glmGamPoi”) using Seurat (v4.3.0) [87]. As there were no batch effects on different replicates, we chose highly variable genes (HVGs) across samples through SelectIntegrationFeatures() to renormalize each sample individually with the same method, and combined all samples via merge(). To reduce the dimensionality of the data, we performed RunPCA() to select the optimal suitable number of principal components (PCs) with a cumulative variance contribution ratio higher than 80%. And then we used FindNeighbors() to construct a k-nearest neighbors (KNN) graph based on selected PCs. For unsupervised clustering of spots, we used the Louvain algorithm via FindClusters() with a resolution from 0.1 to 2.0, and projected data to a two-dimensional (2D) plane via RunUMAP() for visualization. Considering noise spots and rare cell types, we further performed subclustering for each cluster at resolutions from 1.0 to 2.0, retaining clusters with significant biological features, while noise clusters were excluded in the later analysis.

To annotate each cluster, we first collected a set of classical lung cell type markers from published databases [88,89], including their classical cell signatures and abundances. We first divided the clusters into five main groups: alveolar epithelium, bronchial epithelium, vascular endothelium, vascular fibroblast, mesenchyme, and others. Immune cells were not excluded from analysis, but were not resolved into distinct clusters due to low abundance in prenatal stages and technical sensitivity limits. We then reannotated each main group through high-resolution clustering, cell type features enrichment, and spatial distribution. Considering that the resolution of ST data is not exactly single-cell, we annotated each cluster using gene sets via AddModuleScore() rather than individual genes. To verify the rationality of annotation, Pearson correlations were calculated between each cluster via cor() in R, and the results showed strong biological correlations within sub-clusters. To find differentially expressed genes (DEGs) for each annotated cluster, we performed the Wilcoxon rank-sum test for each gene across clusters via FindAllMarkers(logfc.threshold = 0.3, min.pct = 0.15), and the DEGs of each cluster corresponded well to its biological features. In total, we annotated 24 clusters, each with its own unique biological features and a corresponding spatial distribution.

### Spatial deconvolution of spatial RNA data

To obtain the exact cell type proportions of each spot, we used both spacexr (v2.2.0) [90] and cell2location(v0.1.3) [19] to deconvolute each spatial slice. To obtain a comprehensive single-cell reference of mouse lung development, we downloaded the public scRNA-seq dataset [10] and reannotated it by time point, including E12, E15, E16, E18, P0, and P3 separately. To deconvolute spatial spots rationally, we prepared single-cell reference and spatial datasets correspondingly according to the time point.

RCTD from spacexr can learn cell type profiles from scRNA-seq datasets to label ST spots as cell types. Additionally, RCTD includes a platform effect normalization step, which normalizes the scRNA-seq cell type profiles to match the platform effects of the ST dataset. We used raw count matrices as input for both single-cell reference and spatial datasets, and ran RCTD in “full mode” with default parameters. We further processed the output results to visualize cell proportions of each spot in a pie chart via geom_pie().

The cell2location is a principled Bayesian model that can estimate which combination of cell types in which cell abundance could have given the mRNA counts in the spatial data while modelling technical effects. Similarly to RCTD, we used raw count matrices to estimate the absolute spatial abundance of cell types following the official tutorial of cell2location. Additionally, we set “N_cells_per_location = 4” according to the spatial distribution of DBIT-seq spots, and other parameters of cell2location were kept at their default values.

### Spatial ATAC-seq data pre-processing and quality control

We first trimmed adapters of sequence reads using Cutadapt (v4.2) [83] and Trimmomatic (v0.39) [91], and then used a custom Python script to debarcode. Fragment files and feature barcode matrices were generated using scATAC-pro (v1.3.0) [92] based on the reference genome (GRCm38).

We filtered out valid spots similar to spatial RNA data pre-processing, and the filtered matrices were then read into ArchR (v1.0.2) as a tile matrix using createArrowFiles() and ArchRProject(). Furthermore, low-quality spots with a lower than 4 transcription start site (TSS) score and fewer than 1000 fragments were dropped before downstream analysis. Finally, we identified spots that were valid in both spatial RNA and ATAC, and generated fragment files into Signac (v1.13.0) using CreateFragmentObject(), FeatureMatrix(), and CreateChromatinAssay(). Peak sites and P2G links were then predicted through RegionStats() and LinkPeaks(), respectively.

### Spatial ATAC-seq data normalizing, integrating, clustering, and coembedding with RNA-seq data

We firstly combined the peaks of two E12.5 replicates based on tutorial of Signac (v1.13.0), and then recreated Signac-object via CreateFragmentObject(), FeatureMatrix(), and CreateChromatinAssay() using unified peaks. The RunTFIDF() was used to normalize the merged spot–peak matrix, and FindTopFeatures() was applied to choose highly variable peaks (HVPs). To reduce the dimensionality of the data, we performed RunSVD() based on the HVPs previously called. And then, we used FindNeighbors() to construct a KNN graph based on selected Latent Semantic Indexings (LSIs). Finally, FindClusters() with resolution from 0.1 to 2.0 was applied to perform unsupervised clustering, and RunUMAP() was applied to project data to a 2D plane for visualization.

To project both RNA and ATAC into a single embedding, we used FindMultiModalNeighbors() in Seurat (v.4.3.0) to construct a weighted nearest neighbor (WNN) graph by combining the dimensional reductions computed for both modalities. To identify clusters based on co-embedding, we further generated a shared nearest neighbor graph via FindNeighbors() and performed unsupervised clustering via FindClusters(). For two-dimensional visualization of the multi-omics data, the UMAP space was projected via RunUMAP() based on co-embedding.

### TF analysis of E12.5 lung ATAC-seq data

The SCENIC+ (v1.0.0) [22,93] pipeline was applied to predict TFs with related target genes and quantify both TF activity and region enrichment at E12.5, using combined RNA and ATAC data.

We used pycisTopic to detect candidate enhancer regions based on cell type-specific different chromatin accessibility regions (DARs) and topics, and then potential TF binding sites (TFBSs) in candidate enhancers were identified by motif enrichment analysis. To construct the TF–gene regulatory network, we utilized GRNBoost2 to quantify the importance of TFs and candidate enhancers to target genes and inferred the direction of regulation (activation/inhibition) using linear correlations. We then visualized the TF–gene regulatory network via plot_networkx() to identify the key regulators. Finally, we combined motif enrichment analysis results with GRNBoost2 inference to recover the optimal TF for each group of motifs and generate eRegulon.

To screen for significant eRegulons, we filtered out activated eRegulons with *rho* > 0.4, then selected eRegulons in which both TFs and target genes were should be significant at three levels: RNA, ATAC, and spatial.

### Peak-to-gene linkage analysis

To identify putative regulatory relationships between peaks (ATAC) and genes (RNA), we calculated correlations of peak accessibility and gene expression using ArchR (v1.0.2) with the parameters addPeak2GeneLinks(reducedDims = “LSI_Combined”, dimsToUse = 1:60). The function firstly imputed the gene expression values based on the anchor-weight matrix. Next, the function generated cell groups via a *k*-nearest neighbor algorithm to reduce noise and enhance robustness, and groups with overlap greater than 80% with any other were removed. Finally, we visualized the selected correlation between peaks and genes through plotPeak2GeneHeatmap (corCutOff = 0.3). To mark selected peaks and genes, we further predicted motifs of each TF through the AddMotifs() of Signac (v1.13.0) with the database of JASPAR2024. Finally, we generated the heatmap showing significant peak-to-gene linkages at E12.5, as shown in Figure 2A, and we then marked peak-to-gene linkages that overlapped with the motif regions of *Foxf1*, *Nkx2-1*, and *Foxa2* using the Heatmap() and rowAnnotation() of ComplexHeatmap (v2.20.0).

### Moran’s I for spatial auto-correlation

Moran’s I is widely used to evaluate spatial auto-correlation for individual features, and to evaluate spatial auto-correlation between two features, we adapted the extended Moran’s I [43].


$$\mathrm{Global}\;\mathrm{Moran}\prime s\;I=\frac{\sum_{i}\sum_{j}w_{ij}\left(x_{i}-\bar{x}\right)\left(y_{i}-\bar{y}\right)}{\sqrt{\sum_{i}{\left(x_{i}-\bar{x}\right)}^{2}}\sqrt{\sum_{i}{\left(y_{i}-\bar{y}\right)}^{2}}}$$

where *x_i_* and *y_i_* denote the normalized continuous variables of spot*_i_* and spot*_j_*, and *w_ij_* denotes the spatial weight of spot*_i_* and spot*_j_*, which is calculated based on the radial basis function (RBF) kernel with an element-wise normalization.

In this study, we applied Moran’s I to illustrate the dynamic spatial auto-correlation between AT1 and AT2 epithelial cells during development.

### Linear regression between AT1 and AT2 alveolar epithelial cells

To validate the spatial distribution of AT1 and AT2 alveolar epithelial cells during development, we used ggplot2 (v3.4.4) to generate the scatter plot of alveolar epithelial cells at E16.5 and E18.5, and then applied a linear model to fit RNA expression of each spot.

### Airway trajectory inference analysis

To derive the pseudotime of airway cells based on transcriptome similarity, we used Monocle2 (v2.26.0) [94] to project data into a reduced-dimensional space while constructing a principal tree that passes through the middle of the data simultaneously, which is based on reversed graph embedding (RGE). We applied reduceDimension(residualModelFormulaStr = “∼nCount_Spatial + nFeature_Spatial”) to remove technical effects before dimensionality reduction and clustering. For detecting key driver genes that influence cell trajectory, we screened the DEGs with “avg_log_2_FC > 0.35 and p_val_adj < 0.03”, and then generated the pseudotime heatmap using pheatmap (v1.0.12).

### Alveolar trajectory inference analysis

To reconstruct the developmental trajectory of alveolar cells, we utilized STREAM (v1.1) [58] to approximate the gene expression matrix in a structure called the principal graph, which is a set of curves that naturally describe the cell-pseudotime, trajectories, and branching points. In detail, we firstly constructed the principal graph through seed_elastic_principal_graph(clustering = “kmeans”, n_clusters = 12, use_vis = True) based on recomputed original UMAP embedding, and then smoothed the trajectory through elastic_principal_graph(epg_alpha = 0.02, epg_mu = 0.1, epg_lambda = 0.01). A pseudotime heatmap was generated following the same protocol as for the airway trajectory.

To further verify the robustness of the developmental trajectory of alveolar cells, we applied scVelo (v0.2.5) [62] to recover the directed dynamic information by leveraging splicing kinetics, and the resulting of velocity is highly consistent with the trajectory revealed by STREAM.

### Spatial cell–cell communication analysis

For ST data, SpatialDM (v0.1.0) [43] was used to explore the spatial cell–cell communication across slices. We filtered out LR pairs of CellChatDB.mouse, whose ligands and receptors are the same as each other, and retained 1998 valid LR pairs in three types of interactions: “Secreted Signaling”, “ECM–Receptor”, and “Cell–Cell Contact”. To calculate the proper distance matrix for different resolutions, we modified the parameters of “weight_matrix” based on the maximum center-to-center interaction distance (200 μm). The spatialdm_global(n_perm = 1000, method = “z-score”) was used to identify significantly interacting LR pairs in the whole slice, and then the spatialdm_local(n_perm = 1000, method = “z-score”) was used to identify locally significant spots for each LR pair.

In total, SpatialDM provided us with spatially significant LR pairs in each slice, which could be inspected manually based on the image. To identify cluster-specific LR pairs across slices, we applied differential analysis on each annotated cluster based on “−log_10_(local_z_p)”, and generated the cluster-specific LR pairs’ enrichment heatmap using Single-Cell Pipeline (SCP) (v0.5.1). Although the results of SpatialDM did not accurately correspond to cell clusters derived from single-cell expression profiles, we further performed cell–cell communication on the scRNA-seq reference to provide more precise cell cluster information.

### Single-cell RNA cell–cell communication analysis

For scRNA-seq data, CellChat (v1.6.1) [95] was used to explore the cell–cell communication within clusters. To maintain the consistency of cell–cell communication results between scRNA-seq and ST data, we applied the same LR database to CellChat. Firstly, we used computeCommunProb(type = “triMean”, trim = 0.1) to calculate the communication probability between clusters, and then aggregated the results through aggregateNet(thresh = 0.05).

### Single-cell spatial cell–cell communication of alveolar microenvironment

For cell clusters with discrete spatial distributions, we preferentially extracted cluster-specific LR pairs based on CellChat results. Regarding developmentally dynamic cell–cell communication in the alveolar microenvironment, we screened LR pairs specific to the alveolar microenvironment-related cell clusters using CellChat results, then filtered out LR pairs that were not significant in both CellChat and SpatialDM, and finally generated the enrichment heatmap of collagen using SCP (v0.5.1).

### TE quantification and analysis

We utilized scTE (v1.0) [96] to quantify TE expression from ST data. Firstly, we built the genome index for scTE from genecode M21 (GRCm38.p6), and then we put aligned sequence reads [in Binary Alignment/Map (BAM) format] into scTE for TE quantification. All steps were performed in accordance with the official tutorial (https://github.com/JiekaiLab/scTE). To identify cluster-specific TEs, we combined TE profiles of different slices using the same protocol for ST data and then applied differential analysis on each annotated cluster based on TE expression. Finally, we identified 240 cluster-specific TEs (126 LTRs, 66 LINEs, 28 SINEs, 17 DNA elements, and 3 satellite elements) through FindAllMarkers.

### Analysis of alveolar niche before/after birth

We selected alveolar regions of E18.5, P0, and P3, respectively, according to the manual annotations above, and each region was approximately 10 × 10 spots. The principle was that most of the regions were annotated as cell types related to the alveolar microenvironment. To ensure the robustness of the analysis results, we selected 3 regions in each slice.

To identify the differential genes with gradient changes before and after birth in the alveolar regions, we performed DEGs analysis of E18.5 *vs*. P0, E18.5 *vs*. P3, and P0 *vs*. P3. Then, the intersection of each group of upregulated DEGs was defined as the upregulated gene set before birth; conversely, the intersection of each group of downregulated DEGs was defined as the upregulated gene set after birth.

## Ethical statement

Procedures were approved by the Institutional Animal Care and Use Committees of Guangzhou National Laboratory, China (Approval No. GZLAB-AUCP- 2022-10-A05).

## Code availability

All codes used for data analysis are available at GitHub (https://github.com/EddieLv/Temporal-and-spatial-development-of-mouse-lung) and BioCode at the National Genomics Data Center (NGDC), China National Center for Bioinformation (CNCB) (BioCode: BT007890), which are publicly accessible at https://ngdc.cncb.ac.cn/biocode/tool/BT007890.

## Supplementary Material

qzaf053_Supplementary_Data

## Data Availability

All spatial data from this study have been deposited in the Genome Sequence Archive [97] at the NGDC, CNCB (GSA: CRA014010), and are publicly accessible at https://ngdc.cncb.ac.cn/gsa.

## CRediT author statement

**Mingyue Chen:** Data curation, Formal analysis, Investigation, Methodology, Validation, Visualization, Writing – original draft. **Junjie Lv:** Data curation, Formal analysis, Investigation, Methodology, Software, Visualization, Writing – original draft. **Qiao Zhang:** Data curation, Methodology. **Qian Gong:** Investigation. **Ting Zhao:** Methodology. **Zhenping Chen:** Investigation. **Haishen Xu:** Data curation. **Nan Zhou:** Investigation. **Shan Jiang:** Methodology. **Jian Du:** Conceptualization. **Xuepeng Chen:** Conceptualization, Formal analysis, Funding acquisition, Methodology, Project administration, Resources, Supervision, Writing – review & editing. **Yuwen Ke:** Conceptualization, Formal analysis, Project administration, Supervision, Writing – review & editing. All authors have read and approved the final manuscript.
