# Supplementary material for: Spatial Transcriptomics Unveils the Blueprint of Mammalian Lung Development
Source: Genomics Proteomics Bioinformatics. 2025 Jun 10;24(1):qzaf053. doi: 10.1093/gpbjnl/qzaf053 (PMC13466558; doi:10.1093/gpbjnl/qzaf053)
Supplement: Supplementary file 1 [file qzaf053_supplementary_data.zip › Supplementary Material Captions.doc]

**Supplementary material**

**Table S1 PCR oligo and DNA barcode sequences for spatial RNA-seq and ATAC-seq library preparation**

**Table S2 The summary of spatial data in developing mouse lungs**

**Table S3 Identified marker genes for spatial transcriptomic spot clusters in mouse lungs**

**Table S4 Significantly enriched LR pairs according to spatial transcriptome of developing mouse lungs**

**Table S5 The sequences of RNA fluorescence *in situ* hybridization probes**

**Figure S1 Data quality and characterization of the spatial transcriptomic atlas during mouse lung development**

**A**. Summary of the resolution and spatial library construction methods for each tissue section. **B**. Representative immunofluorescence staining images for the epithelial cell marker NKX2.1 and the smooth muscle cell marker ACTA2 in developing mouse lungs from E12.5 to P14. Scale bars: 100 μm. The zoomed-in regions are showed on the right. scale bars: 20 μm. **C**. UMAP visualization of all modified DBiT-seq profiled spots (20 μm resolution) stratified by developmental stages. **D**. UMAP visualization of E12.5 (left), E16.5 (middle), and E18.5 (right) modified DBiT-seq profiled spots (20 μm resolution) stratified by biological replicates. **E**. Dot plot illustrating the normalized gene expression levels across 24 spatial clusters. Dot size represents the percentage of expressed genes. Genes marked with an asterisk (*) are canonical markers, while the remaining genes are newly identified in this study. **F** and **G**. Normalized spatial gene expression patterns of cluster marker genes: *Wt1* **(F)** and *Vegfc* **(G)** on ST sections. Scale bars: 200 μm. MISAR-seq, microfluidic indexing-based spatial assay for transposase-accessible chromatin and RNA-sequencing; DAPI, 4',6-diamidino-2-phenylindole.

**Figure S2 Single-cell mapping and functional enrichment analysis of spatial clusters**

**A** and **B**. UMAP embeddings of the two published scRNA-seq datasets (PMID: 34927678 and 33707239). **C** and **D**. Heatmaps displaying the distribution of scaled cell type abundances across spatial clusters for the two scRNA-seq datasets (PMID: 34927678 and 33707239). **E**. Table summarizing enriched scRNA-seq cell types for the 24 spatial clusters (20 μm resolution data) and the manually curated spatial annotations used in this study. **F**. Bar plots of GO functional enrichment for sub-clusters of airway, mesenchymal, and vascular regions. The red dotted line indicates the significance cutoff (*P* value = 0.05). GO, Gene Ontology.

**Figure S3 Spatial annotation and deconvolution analysis of the subcellular-resolution Stereo-seq data**

**A**. Violin plots displaying the distribution of nUMI and nGene across different resolutions of P14 Stereo-seq data. The bin100 (50 μm) was selected for downstream analyses. **B**. Spatial visualization of P14 data, annotated based on unsupervised clustering results. Scale bars: 200 μm. **C**. Heatmaps illustrating the distribution of scaled cell type abundances across spatial clusters for two scRNA-seq datasets (PMID: 34927678 and 33707239). **D**. Spatial mapping of scRNA-seq-derived cell type abundances (ciliated, AT2, mesothelium, VSMC, lymphatic EC, and gCap) (PMID: 34927678) on P14 data using cell2location. Scale bars: 200 μm. **E**. Spatial mapping of scRNA-seq-derived cell type abundances (ciliated, AT2, mesothelium, VSMC, lymphatic EC, and immune cells) (PMID: 33707239) on P14 data using cell2location. Scale bars: 200 μm. VSMC, vascular smooth muscle cell; EC, endothelial cell; ASMC, airway smooth muscle cell; aCap, aerocyte.

**Figure S4 Spatial transcriptomic data visualization and spot-cluster deconvolution during early mouse lung development**

**A**. Reclustering UMAP of 20 μm resolution E12.5 ST data (E12.5_1 and E12.5_2), revealing 13 distinct spot clusters. **B**. UMAP visualization of reanalyzed E12 scRNA-seq data (PMID: 34927678). **C**. Spatial visualization of reannotated spot clusters at E12.5 (left). Deconvolution using RCTD to map cell type proportions from scRNA-seq data onto each spot (right). Zoomed-in views of airway regions for the E12.5_1 and E12.5_2 ST sections (middle). Scale bars: 200 μm. **D**. Spatial mapping of deconvoluted RCTD weights for Sox2+ early epithelium and Sox9+ early epithelium on the two replicate E12.5 ST sections. Scale bars: 200 μm. **E**. Spatial mapping of cell2location abundance scores for Sox2+ early epithelium and Sox9+ early epithelium on the two replicate E12.5 ST sections. Scale bars: 200 μm. nSpots, number of ST spots; nCells, number of cells.

**Figure S5 Quality control, clustering, and mapping results of ATAC-seq and RNA-seq data from E12.5 spatial multi-omics**

**A**. Violin plots displaying FRiP, nPeaks, nFrags, nUMI, and nGene across two 10 μm resolution E12.5 slices (E12.5_3 and E12.5_4). **B**. Density plots illustrating the distribution of log_10_(nFrags) and TSS enrichment scores for E12.5_3 (left) and E12.5_4 (right). **C–E**. Reclustering UMAP of two E12.5 slices, with spots labeled by replicates (top) and cluster annotations (bottom), separately for RNA-seq **(C)**, combined data **(D)**, and ATAC-seq **(E)**. **F**. Sankey plot demonstrating the correlation between RNA-seq, ATAC-seq, and combined data annotations. FRiP, fraction of reads in peaks; nPeaks, number of peaks; nFrags, number of fragments; TSS, transcription start site.

**Figure S6 Gene regulatory networks of epithelial clusters in early mouse lung development**

**A**. Spatial visualization of RNA clusters on E12.5_3 and E12.5_4 sections using modified MISAR-seq data. Scale bars: 200 μm. **B**. Heatmaps displaying the distribution of scaled cell type abundances across spatial clusters for the scRNA-seq dataset (PMID: 34927678). **C**. Heatmap/dot plot illustrating the z-score TF expression (color scale) and cell-type specificity (RSS, size scale) of TF-eRegulons enriched in **epithelial clusters**. **D** and **E**. TF–region–gene regulatory networks for SMO-C2 **(D)**, and SMO-C3 **(E)**. Dashed lines represent TF–region regulatory relationships, while solid lines indicate region–gene regulatory interactions.

**Figure S7 Spatial visualization of epithelial clusters-enriched TFs and their target genes during early mouse lung development**

**A**. Spatial mapping of spot cluster-specific TF-eRegulons, showing TF expression, gene AUC RSS, and region AUC RSS for SMO-C1 (Sox2+ epithelial progenitors-enriched; *Foxa2*). Scale bars: 200 μm. **B**. H&E staining of the adjacent slice of E12.5_3 and spatial distribution of TF target genes on the E12.5_3 section. Target genes include *Ap1s3* for *Foxa2*, *Lin7a* for *Nkx2-1*, and *Sox2* for *Grhl2*. Scale bars: 200 μm. **C** and **D**. Spatial mapping of spot cluster-specific TF-eRegulons, showing TF expression, gene AUC RSS, and region AUC RSS for **(C)** SMO-C3 (Sox9+ epithelial progenitors-enriched; *Nkx2-1*), and **(D)** SMO-C1/C3 (Sox2+ and stalk epithelial cells-enriched; *Grhl2*). Scale bars: 200 μm. **E.** Genome browser tracks displaying chromatin accessibility (top), peak sites, Signac-derived Peak2GeneLinks, SCENIC+ predicted eRegulonLinks (middle), gene tracks (bottom), and corresponding gene expression (right panel) for TF-eRegulon–target gene pairs: (*Grhl2* + *Foxa1*)–*Sox2*.

**Figure S8 Gene regulatory networks and spatial visualization of mesenchymal clusters in early mouse lung development**

**A**. Heatmap/dot plot illustrating the z-score TF expression (color scale) and cell-type specificity (RSS, size scale) of TF-eRegulons enriched in **mesenchymal clusters**. **B**. TF–region–gene regulatory networks for SMO-C6. Dashed lines represent TF–region regulatory relationships, while solid lines indicate region–gene regulatory interactions. **C**. Spatial mapping of spot cluster-specific TF-eRegulons, showing TF expression, gene AUC RSS, and region AUC RSS for SMO-C6 (airway smooth muscle cell precursors; *Foxf1*). Scale bars: 200 μm. **D.** Genome browser tracks displaying chromatin accessibility (top), peak sites, Signac-derived Peak2GeneLinks, SCENIC+ predicted eRegulonLinks (middle), gene tracks (bottom), and corresponding gene expression (right panel) for TF-eRegulon–target gene pairs: *Map3k20*–*Foxf1*. **E**. Spatial visualization of target gene *Map3k20* for *Foxf1* on the E12.5_3 section. Scale bars: 200 μm.

**Figure S9 Spatial cell–cell communication during mouse lung development analyzed by SpatialDM**

**A**. Scatter plots illustrating LRs enrichment in each sample using SpatialDM. The X-axis represents Log_2_(global_I + 1), and the Y-axis represents −Log_2_(z_pval + 1). **B**. Example scatter plot showing the correlation of LR enrichment between two biological replicates at E16.5. **C**. Heatmap displaying Pearson’s correlation coefficients for shared LRs across different samples. **D**. Top: H&E staining of adjacent tissue sections. Middle: spatial visualization of *Bmp8b*–(*Bmpr1b*+*Acvr2b*) LR spatial autocorrelation probability (1 − z_pval) in airway structures. Bottom: spatial distribution of spot clusters within the bronchi group across different lung sections during development. Top right: histogram showing the proportion of *Bmp8b*–(*Bmpr1b*+*Acvr2b*) interactions in different lung structure spots from E12.5 to P3. Scale bars: 200 μm. **E**. Top: H&E staining of adjacent tissue sections. Middle: spatial visualization of *Col1a2*–(*Itga11*+*Itgb1*) LR spatial autocorrelation probability (1 − z_pval) in airway and vascular structures. Bottom: spatial distribution of spot clusters within the bronchi and vessel groups across different lung sections during development. Right panel: histogram showing the proportion of *Col1a2*–(*Itga11*+*Itgb1*) interactions in different lung structure spots from E12.5 to P3. Scale bars: 200 μm. fdr, false discovery rate.

**Figure S10 Spatial development of airway epithelial clusters in the mouse lung**

**A**. Selection of lung bronchial airway spots based on manual annotation and morphological characteristics from E12.5, E16.5, E18.5, P0, and P3 lungs. **B**. Reclustering UMAP of selected lung bronchial airway spots. AW: airway. **C**. Heatmaps displaying the distribution of scaled cell type abundances across spatial clusters for the scRNA-seq datasets (PMID: 34927678). **D**. Spatial gene expression patterns of classical markers for different airway epithelial cell types, including *Spag17* (ciliated cells), *Scgb3a2* (secretory cells), *Pcsk1* (PNECs), *Myh11* (ASMCs), and *Mki67* (proliferation marker). Red dotted lines mark airways, and yellow dotted lines mark vessels. Scale bars: 200 μm. **E**. Bar plot illustrating the dynamic proportions of airway epithelial clusters during lung development. **F**. Developmental trajectory of the four airway epithelial clusters. **G**. Visualization of gene expression for classical proximal epithelial progenitor marker (*Sox2*) and PNEC marker (*Ascl1*) along the airway developmental trajectory. PNECs, pulmonary neuroendocrine cells.

**Figure S11 Spatial distribution of vessel-associated clusters during mouse lung development**

**A**. Reclustering UMAP visualization of vascular spots (10 μm and 20 μmresolution ST data). **B**. Heatmaps displaying the distribution of scaled cell type abundances across vessel-associated spatial clusters for the scRNA-seq datasets (PMID: 34927678). **C**. Heatmap showing normalized gene expression of DEGs for six spot clusters of mouse lung vascular cells. **D**. Normalized spatial gene expression patterns of cluster marker genes on ST sections, including *Trpc6* (pericyte marker) and *Kit* (gCap cell marker). Red dotted lines on H&E staining mark airways, and black dotted lines mark large vessels adjacent to airways. Scale bars: 200 μm. DEGs, differentially expressed genes.

**Figure S12 Spatial developmental trajectories of alveolar epithelial clusters during mouse lung development**

**A**. Reclustering UMAP visualization of spots in the distal airway or alveolar niche from E12.5_1 (20 μm), E12.5_2 (20 μm), E16.5_3 (10 μm), and E18.5_3 (10 μm) sections. **B**. Heatmaps displaying the distribution of scaled cell type abundances across alveolar spatial clusters for the scRNA-seq dataset (PMID: 33707239). **C**. Separated developmental trajectories showing reannotated clusters (left), actual developmental stages (mMiddle), and pseudotime states (right). **D**. RNA velocity trajectory of alveolar epithelial clusters based on the original UMAP embedding, analyzed using scVelo. **E**. Visualization of gene expression for distal/alveolar cell markers (*Sox9*, *Hopx*, *Ager*, *Sftpc*, and *Sftpb*) along the STREAM trajectory. **F**. Heatmap showing DEGs for alveolar epithelial clusters at different developmental states. STREAM, Single-cell Trajectories Reconstruction, Exploration And Mapping.

**Figure S13 The spatial convergence of AT1 and AT2 cells mediated by collagen signaling pathways during the canalicular–saccular transition**

**A**. Scatter plots showing cell2location abundance scores for AT1 and AT2 cells at E16.5 (left) and E18.5 (right). Linear regression was applied to assess correlations. **B**. RNA fluorescence *in situ* hybridization confirming the spatial expression of *Ager* and *Sftpc* at E16.5 (t) and E18.5 (bottom). White dashed lines indicate *Ager*+*Sftpc*+ airways, and red dashed lines outline *Ager*−*Sftpc*+ airway tips. Scale bars: 20 μm. **C**. Heatmap showing the probability of LR interactions from the collagen pathway within the alveolar niche. All LRs are significant in both scRNA-seq CellChat analysis and at least one ST section. **D**. Circle plot illustrating LRs involving myofibroblast cells as main receptor-expressing populations in collagen signaling pathways, derived from scRNA-seq data (PMID: 34927678). Labels are consistent with Figure 4E. **E**. Circle plot illustrating LRs involving (proliferating) gCap cells as main receptor-expressing populations in collagen signaling pathways, derived from scRNA-seq data (PMID: 34927678). Labels are consistent with Figure 4E. **F**. Example showing the spatial enrichment of *Col4a4*–*Sdc4* LR in alveolar epithelial cells and regions in E16.5 and E18.5 mouse lungs. **G** and **H**. Immunofluorescence staining for COL4A4 and SDC4 proteins at E16.5 **(G)** and E18.5 **(H)**. COL4A4 and SDC4 co-staining with AGER was performed on two adjacent sections, respectively. Co-localization of COL4A4 and SDC4 proteins was observed at E16.5 (white dotted lines) and E18.5. Scale bars: 200 μm.

**Figure S14 Molecular characteristics of the alveolar region before and after birth**

**A**. Selected lung alveolar niche spots according to manual annotation and morphological characteristics from 20 μm resolution E18.5, P0, and P3 data. **B**. The heatmap showing transitional alveolar niche specific genes during mouse lung alveolar maturation (E18.5, P0, and P3). **C**. Bar plots of pathway enrichment analysis for alveolar niche specific genes before (top) or after (bottom) birth. **D**. The spatial highlighting of normalized gene expression level for downregulated genes after birth (*Hif3a*, *Fgf10*, *Fn1*, and *Rtkn2*) across E18.5, P0, and P3. **E**. The spatial highlighting of normalized gene expression level for upregulated genes after birth (*Tgfbi* and *Bmpr2*) across E18.5, P0, and P3. KEGG, Kyoto Encyclopedia of Genes and Genomes; BP, biological process.
